# Mimicking Photosystem I with a Transmembrane Light Harvester and Energy Transfer‐Induced Photoreduction in Phospholipid Bilayers

**DOI:** 10.1002/chem.202003391

**Published:** 2020-12-21

**Authors:** Andrea Pannwitz, Holden Saaring, Nataliia Beztsinna, Xinmeng Li, Maxime A. Siegler, Sylvestre Bonnet

**Affiliations:** ^1^ Leiden University Leiden Institute of Chemistry Einsteinweg 55, 2333 CC Leiden The Netherlands; ^2^ Johns Hopkins University Department of Chemistry Maryland 21218 Baltimore USA

**Keywords:** electron transfer, energy transfer, phospholipid bilayers, photoreduction, vesicles

## Abstract

Photosystem I (PS I) is a transmembrane protein that assembles perpendicular to the membrane, and performs light harvesting, energy transfer, and electron transfer to a final, water‐soluble electron acceptor. We present here a supramolecular model of it formed by a bicationic oligofluorene **1^2+^** bound to the bisanionic photoredox catalyst eosin Y (EY^2−^) in phospholipid bilayers. According to confocal microscopy, molecular modeling, and time dependent density functional theory calculations, **1^2+^** prefers to align perpendicularly to the lipid bilayer. In presence of EY^2−^, a strong complex is formed (K_a_=2.1±0.1×10^6^ m ^−1^), which upon excitation of **1^2+^** leads to efficient energy transfer to EY^2−^. Follow‐up electron transfer from the excited state of EY^2−^ to the water‐soluble electron donor EDTA was shown via UV–Vis absorption spectroscopy. Overall, controlled self‐assembly and photochemistry within the membrane provides an unprecedented yet simple synthetic functional mimic of PS I.

## Graphical abstract

**Mimicking PS I**: Light absorption, energy transfer and electron transfer were obtained in a supramolecular mimic of photosystem I.

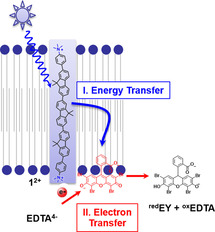

## Introduction

In nature, photosynthetic organisms absorb sunlight to convert it into high‐energy chemicals used as bioenergy carriers. In order to do so, they arrange several protein super complexes with precisely oriented chromophores in phospholipid membranes.[^1^, ^2^, ^3^] One example is photosystem I (PS I) which is surrounded by multiple units of the protein light harvesting complexes I (LHC I) to harvest sunlight in the UV and visible range of the solar spectrum to funnel the photon energy to the reaction center in photosystem I (PS I).^[1]^ Light energy transfer within the membrane is enabled by orientation control of numerous light harvesting chromophores within the membrane and with respect to the energy accepting reaction center.^[1]^ The reaction center itself is a red light‐absorbing chlorophyll dimer which triggers multistep electron transfer reactions in the phospholipid membrane to a final electron acceptor.[^1^, ^2^, ^3^, ^4^, ^5^] Synthetic self‐assemblies are aimed at mimicking functions of cells and photosynthesis.[^6^, ^7^, ^8^] In particular, phospholipid membranes and vesicles (e.g. liposomes) can serve as a scaffold for mimicking cellular compartmentalization,[^9^, ^10^, ^11^] light harvesting,^[12]^ membrane interactions,[^13^, ^14^] transmembrane electron transfer,[^10^, ^15^, ^16^, ^17^, ^18^, ^19^] and co‐assembly of photosensitizers with electron relays and catalysts.[^20^, ^21^, ^22^, ^23^] In very rare cases the assembly of chromophores at phospholipid membranes enabled for light‐induced energy *and* electron transfer.^[24]^ Self‐assembled transmembrane molecular wires were able to achieve electron transfer across artificial and natural phospholipid membranes, though in the absence of light.[^25^, ^26^, ^27^, ^28^] Liposomes doped with transmembrane electron transferring chromophores coupled to proton and ion transfer lead to pH and concentration gradients across membranes.[^26^, ^27^, ^28^] One common design principle for membrane‐spanning molecules it that they shall comprise both a central hydrophobic and one or two terminal hydrophilic groups. With two end‐groups, the distance between these hydrophilic groups should match the thickness of the lipid bilayer, as distance mismatch tends to lower membrane stability.[^29^, ^30^, ^31^, ^32^, ^33^]

**Scheme 1:**
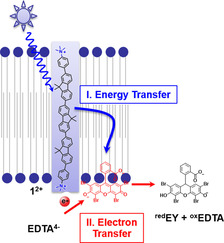
Light absorption by **1^2+^** is followed by energy transfer to eosin Y (EY^2−^, in red) and subsequent electron transfer from the electron donor EDTA^4−^ to the excited EY^2−^.

In this study, we constructed an artificial, biomimetic analogue of photosystem I based on a rigid, oligofluorene chromophore that precisely self‐assembles perpendicularly to phospholipid bilayers. We chose here a rigid, symmetrical oligofluorene core composed of eight conjugated aromatic rings, directly connected to two terminal, hydrophilic trimethylammonium anchoring groups. The designed oligo‐fluorene **1^2+^** is depicted in Scheme 1. The ammonium groups are separated by a distance of 3.5 nm, which fits best with typical thicknesses of phospholipid bilayers (vide infra*)*.^[33]^ Upon light absorption, this oligofluorene funnels the photon energy into an energy acceptor finally capable of transferring electrons at the water‐membrane interface.

## Results and Discussion

**Figure 1:**
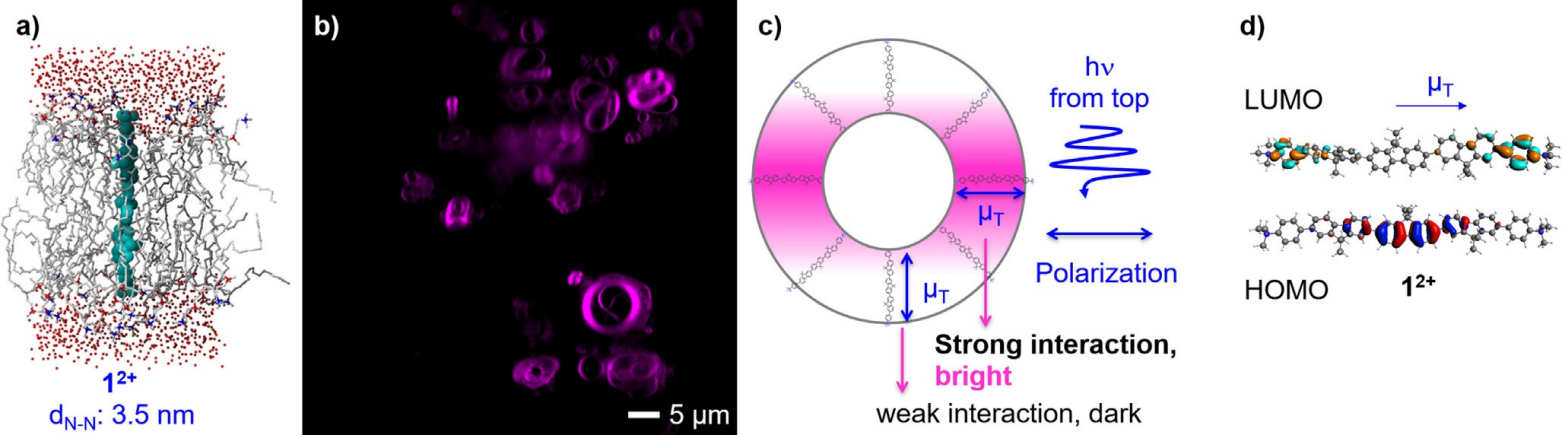
a) Molecular dynamics model of **1^2+^** in a transmembrane geometry in a phospholipid bilayer. Color‐code: **1** ^2+^: turquoise, space filling model; lipid bilayer and water: stick model, red: oxygen, yellow: phosphorous, blue: nitrogen, grey: carbon, green: chloride. Hydrogen atoms are omitted for clarity. b) Confocal luminescence microscopy images of giant DMPC vesicles doped with 1 mol % **1** ^2+^ at pH 7.8, laser excitation at *λ*_ex_=405 nm, detection in the range: 420–514 nm. c) Schematized interaction of the transition dipole *μ*_T_ of **1** ^2+^ with the incident (polarized) laser light exciting the sample from top. d) HOMO, LUMO, and transition dipole moment, of **1^2+^** calculated by TDDFT at the CAM‐B3LYP/TZP level.

The synthesis of **1**(PF_6_)_2_ was performed in four steps described in the Supporting Information. A molecular dynamics model of **1^2+^** in a phospholipid bilayer (Figure 1 a) confirmed that the 3.5 nm distance between the ammonium groups fits ideally with the 1,2‐dimyristoyl‐sn‐glycero‐3‐phosphocholine (DMPC) and 1,2‐dipalmitoyl‐sn‐glycero‐3‐phosphocholine (DPPC) membrane thickness of 3.1–3.4 and 3.4–3.7 nm, respectively.[^34^, ^35^]

**Scheme 2:**
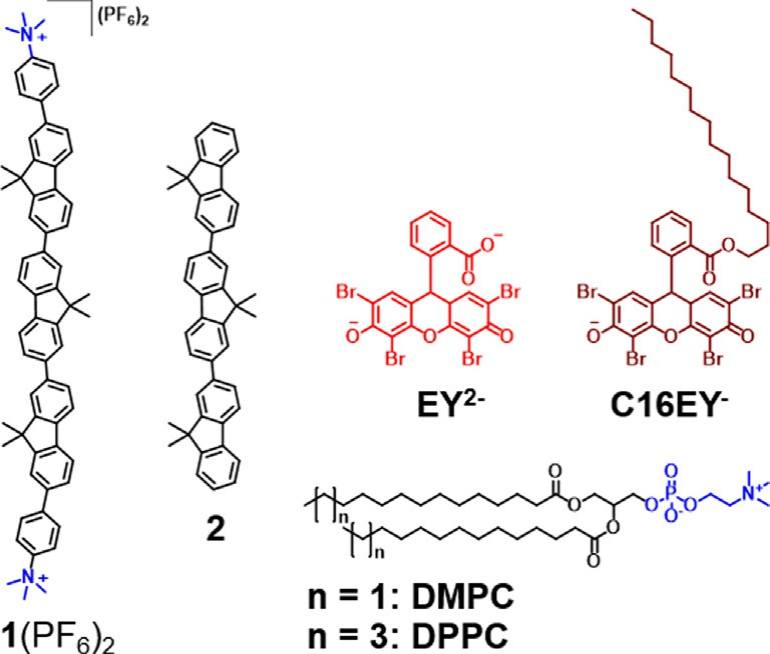
Chemical structures of the chromophores and lipids (DMPC and DPPC) used in this work.

In organic solvent, **1**(PF_6_)_2_ absorbs at 358 nm in methanol, and its hydrophobic core molecule **2** (Scheme 2) absorbs at slightly higher energy in chloroform (349 nm, see Table 1). In spite of their similar emission maxima (≈400 nm) and stokes shifts (48 vs. 44 nm, respectively), the molar absorption coefficient (*ϵ*) of **1^2+^** in methanol was found significantly higher than that of **2** in chloroform (16×10^4^ m ^−1^ cm^−1^ vs. 6.8×10^4^ m ^−1^ cm^−1^) suggesting different types of excited states. Upon incorporation into liposomes neither **1^2+^** nor **2** experienced significant spectroscopic changes compared to organic solvents. Very small shifts of their absorbance maxima might result from Tyndall scattering of the liposomes suspension (Figure S8), while the shift in luminescence upon incorporation into liposomes was hardly measurable (≈2 nm). Such minor spectroscopic variations suggest negligible solvent effects and minor aggregation of **1** ^2+^ and **2** in phospholipid membranes as compared to organic solvent, which differs from other oligovinylene chromophores.[^25^, ^36^]

**Table 1:** Spectroscopic and properties of the investigated compounds.

	Conditions	*λ*_abs_ [nm] (*ϵ* [10^4^ m ^−1^ cm^−1^])	*λ*_em_ [nm]
**1^2+^**	methanol	358 (16)	404; 422
	DMPC vesicles^[a]^	362	404; 425
	TD–DFT (CAMB3LYP)	352	–
**2**	CHCl_3_	349 (6.8)	393; 414
	DMPC vesicles^[a]^	350	393; 413
	TD‐DFT (PB0)	353	–
EY^2−^	water, pH 7.8^[b]^	517	538
	DPPC vesicles^[a,c]^	517‐528	545
C16EY^‐^	methanol	531	556
	DPPC vesicles^[a]^	545	574

[a] DMPC or DPPC, 1 % chromophore and 1–4 % NaDSPE–PEG2K in phosphate buffer, pH 7.8. [b] Phosphate buffer. [c] Dependent on concentration, in line with refs. [38, 39].

Modeling the absorption spectra with time‐dependent density functional theory (TD‐DFT) yielded the lowest energy absorption bands at 352 nm for **1^2+^** and 353 nm for **2** respectively, which is reasonably similar to the experimental values (Table 1). The CAMB3LYP functional was chosen for **1** ^2+^ to take into account the charge transfer (CT) character found for its lowest excited states: As shown in Figure 1 d, the calculated HOMO and LUMO of the ground state of **1^2+^** are located in the middle and at the extremities of the oligofluorene **1^2+^**, respectively. By contrast, the HOMO and LUMO of **2** (Figure S9) are both located at the center of the trifluorene molecule, lowest energy transition is a more classical π–π* character (Figure S9).

In order to see whether **1^2+^** aligns indeed perpendicularly to lipid membranes, confocal microscopy was performed on giant multilamellar vesicles using laser excitation at 405 nm and detection in the region 420–514 nm (Figure 1 b). The luminescence images were superimposable with the simultaneously recorded transmission image (Figure S16), which demonstrates that **1^2+^** is selectively taken up in the lipid bilayer.

For the reference compound **2** no selective staining of the bilayer was observed for **2** under comparable experimental conditions (see Figure S17), which we attribute to preferred π‐stacking of **2** over its solubility in the lipid bilayer structure.

Furthermore, for vesicles with **1^2+^** a double half‐moon shaped emission profile was observed in all vesicles in the microscopic image (Figure 1 b), which is typical for molecules forming a circle in the observation plane.^[25]^

The interaction of each chromophore molecule with the laser beam depends on the orientation of their transition dipole moment with respect to the direction of propagation of the light beam. As the incident laser light is polarized, all molecules with a transition dipole moment (*μ*_T_) parallel to the polarization plane of the laser, absorb more light and therefore exhibit brighter luminescence, which explains the bright regions on the thick parts of both half‐moons. In the thin regions of the image the transition dipole moment of **1^2+^** is orthogonal to the polarization plane, therefore the absorption of the light beam, and hence the luminescence image are weaker. The transition dipole moment of the lowest electronic transition of **1^2+^**, is parallel to the long axis of the molecule (Figure 1 d) and has 6.32 Debye according to TD–DFT calculation at the CAM‐B3LYP/TZP level. Hence, spherically assembled transition dipole moments correspond to spherically assembled molecules.

In principle, one could argue that the half‐moon effect might be due to either a parallel, or a perpendicular (transmembrane) alignment of **1^2+^** with respect to the lipid bilayer. We performed molecular dynamics simulations using Gromacs 2018 software^[37]^ in order to check that. First, the self‐assembly of 6 independent random distributions of 128 DMPC molecules and one molecule of **1**(PF_6_)_2_ in water was modelled for 200 ns, as described in the Supporting Information. In all cases spontaneous bilayer formation was observed, and in four cases out of six **1^2+^** indeed ended up in a transmembrane fashion (see supplementary movie Movie1.mpg), whereas two simulations ended up in a parallel configuration. This result suggested a preference of **1^2+^** for a transmembrane self‐assembly, but it would not be affordable to quantify this preference using this computationally intensive method. Thus, in two of these simulations we computed the binding free energy of **1^2+^** to the membrane, Δ*G*_bind_ either in the transmembrane or in the parallel configuration (see details in the Supporting Information). The averaged Δ*G*_bind_ for the perpendicular (transmembrane) and parallel configuration were −165.5 kJ mol^−1^ and −22.4 kJ mol^−1^, respectively, which further confirmed the preference of **1** ^2+^ for the transmembrane configuration. Overall, these modeling studies supported our design hypothesis, that the half‐moon effect observed in confocal images of giant vesicles containing **1** ^2+^, is due to a preference for a transmembrane configuration of this linear molecule.

In nature, photosystem I transfers the excitation energy of the transmembrane molecular light harvester to a second dye in the membrane, to finally induce charge transfer. To mimic this system eosin Y (EY^2−^) was chosen as a co‐dopant in lipid membranes, because this dye has been widely used in photoelectron transfer^[40]^ and photocatalytic proton and CO_2_ reduction studies on lipid bilayers and cell membranes.[^22^, ^40^, ^41^] Therefore, **1^2+^** and H_2_EY were added in different ratios into the lipid bilayer of DPPC liposomes during lipid film preparation. Deprotonation of H_2_EY to EY^2−^ occurred upon hydration of the lipid films with a phosphate buffer at pH 7.8, as demonstrated by the characteristic absorption maximum at 544 nm for DPPC:**1^2+^**:EY^2−^ liposomes (1000:13:10 n/n/n ratio). Interestingly, this band is significantly red‐shifted compared to homogeneous solution (*λ*_max_=517 nm in water[^42^, ^43^, ^44^]). The absorbance of **1^2+^** was slightly blue‐shifted in presence of EY^2−^ in the membrane, from 356 nm in DPPC:**1^2+^** liposomes (1000:13 n/n ratio) to 351 nm in DPPC:**1^2+^**:EY^2−^ liposomes (1000:13:10 n/n/n ratio). Both shifts are indicative of supramolecular interaction within the membrane between EY^2−^ and **1^2+^** (in the ground state).^[42]^ These interactions were confirmed by molecular dynamics simulations of one molecule of **1^2+^** and one molecule of EY^2−^ in a DMPC lipid bilayer model. Within 30 ns simulation both dyes showed close contact interactions, characterized by a distance of less than 1 nm between the two oppositely charged species. Respective graphical presentations of this model can be found in Figure S6 and Figure S7.

**Figure 2:**
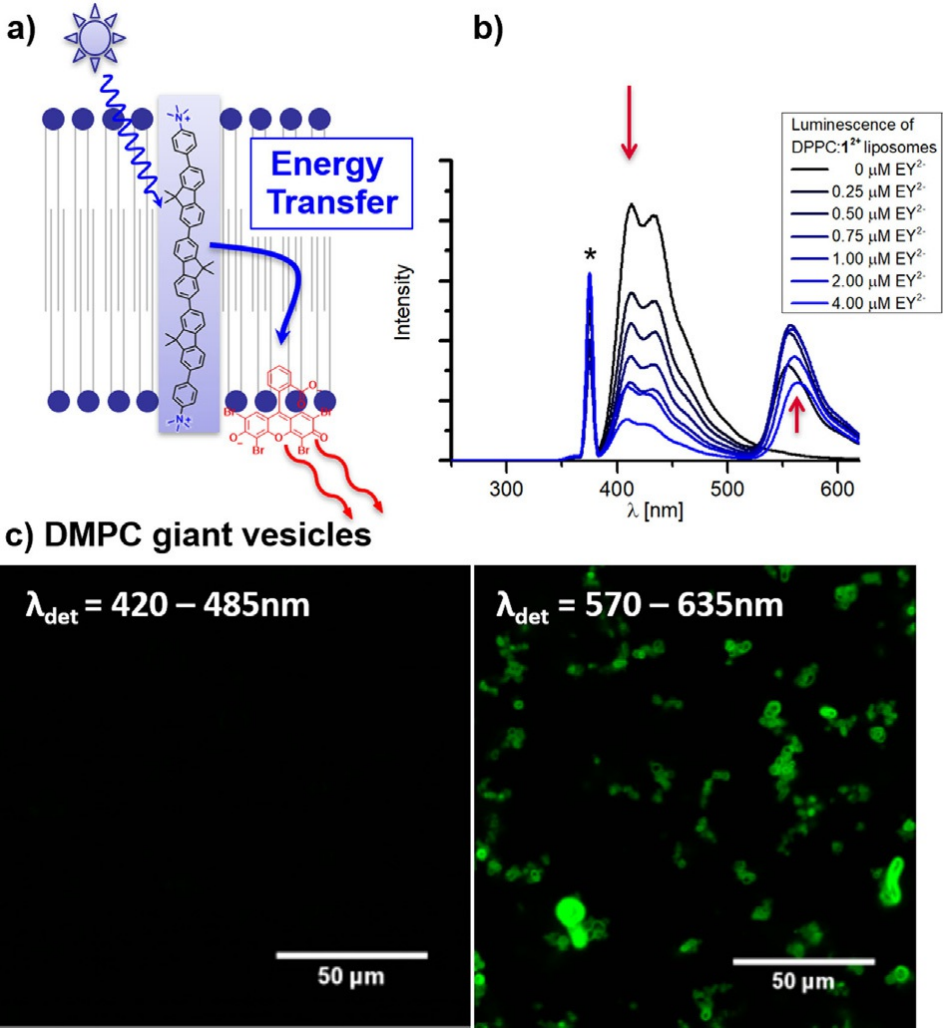
a) Scheme of energy transfer within the phospholipid bilayer. b) Luminescence spectra upon excitation of 1.25 mm liposomes DPPC:**1^2+^**:EY^2−^ at 374 nm at pH 7.8. The liposomes contained 0.3 % NaDSPE–PEG2K, 1.3 % **1^2+^** and various concentrations of EY^2−^ added to the lipid mixture during liposome preparation. The asterisk (*) marks the scattered excitation light. c) Confocal images (excitation at 405 nm) of DMPC:**1^2+^** in presence of 10 μm EY^2−^ added to the solution after vesicle formation at pH 7.8.

The formation of a supramolecular complex between **1^2+^** and EY^2−^ in liposomes was confirmed by efficient energy transfer from **1^2+^** to EY^2−^ observed upon selective photoexcitation of **1^2+^** (at 374 nm) lighting up the emission band of EY^2−^ (Figure 2 b). The steady‐state emission spectrum of such DPPC:**1^2+^**:EY^2−^ liposomes showed gradual quenching of the emission of **1^2+^** at 404 nm upon adding increasing concentrations of EY^2−^ into the membrane, while increasing emission of EY^2−^ was observed (Figure 2 b). Plotting the inverse of the luminescence intensity vs. acceptor concentration in a Stern–Volmer plot indicated combined static and dynamic quenching (Supporting Information, Figure S15). Eq. 1 was used to obtain the association constant (*K*_a_ in M^−1^) for the equilibrium shown in Eq. 2:^[45]^

(1) $$\frac{I_{0}}{I}=\left(1+K_{a}\cdot \left[{EY}^{2-}\right]\right)\cdot \left(1+K_{SV}\cdot \left[{EY}^{2-}\right]\right)$$

(2) $$\mathrm{DPPC}:1{}^{2+}\;+\;\mathrm{EY}{}^{2\text{-}}\;\vec{\leftarrow }\;\mathrm{DPPC}:1{}^{2+}:\mathrm{EY}{}^{2\text{-}}$$

In Eq. (1), *I*_0_ and *I* represent the emission intensity of **1^2+^** in absence and in presence of the quencher [EY^2−^], and K_SV_ the Stern–Volmer constant (in M^−1^) for the dynamic quenching of the emissive S_1_ excited state of **1^2+^** by EY^2−^. In absence of EY^2−^ DPPC:**1^2+^** liposomes had a luminescence lifetime of 1.4 ns. In the lower concentration regime of EY^2−^ ([EY^2−^] <0.5×[**1^2+^**]) the dynamic quenching takes place with a Stern–Volmer constant K_SV_=5.3⋅10^5^ m ^−1^ while the association constant (K_a_) for its static component is K_a_=(2.1±0.1)×10^6^ m ^−1^. This association constant is 3 orders of magnitude stronger than the reported association of EY^2−^ to bare DPPC vesicles at pH 7 (K_a_=(1.0±0.1)×10^3^ m ^−1^)^[39]^ which highlights the strong attracting effect of the positively charged membrane‐doping agent **1^2+^**. At higher concentration of EY^2−^ (0.5<[EY^2−^]/[**1^2+^**]<1) the quenching behavior does not follow the trend of eq. 1 anymore, which might be due to dimerization of EY^2−^ at the membrane interface.^[46]^

Luminescence quenching was also observed by confocal luminescence microscopy of micrometer sized multi‐lamellar giant vesicles. The blue luminescence observed with DMPC vesicles containing **1^2+^** was quenched almost completely upon addition of 10 μm EY^2−^ to the outer aqueous phase of the giant vesicles, while the luminescence of EY^2−^ in the red region of the spectrum was switched on (Figure 2 c). Interestingly, this phenomenon was not observed for apparently similar DPPC:**1^2+^** vesicles. Upon addition of 10 μm EY^2−^ to the outer aqueous phase of these vesicles at room temperature, the luminescence of **1^2+^** was only partly quenched lighting up only parts of the EY^2−^ luminescence. This could be explained by the fact that only the outer shells of the multi‐lamellar vesicles are interacting with EY^2−^. According to the leakage test with DPPC:**1^2+^** (Supporting Information, p. S32), lipid bilayers are impermeable to water‐soluble species. Therefore, inner lamellas of multilamellar vesicles are not affected by quenching via energy transfer. By contrast, DMPC vesicles are inherently leaky and more fluid at room temperature, because their phase transition temperature coincides with room temperature.[^47^, ^48^] Nevertheless, these data underline that the supramolecular complex [**1^2+^**:EY^2−^] forms within the phospholipid bilayer and provides an efficient scaffold for energy transfer from the transmembrane blue‐light harvesting oligofluorene **1^2+^** to the photoredox catalyst EY^2−^.

**Figure 3:**
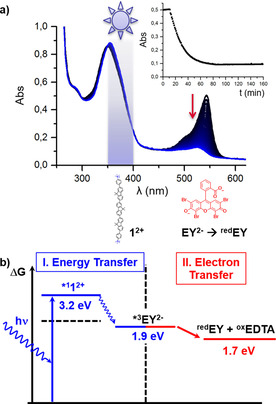
a) Evolution of the UV–Vis absorption spectrum of DPPC:**1^2+^**:EY^2−^ liposomes containing 0.3 % NaDSPE–PEG2K and 1.3 % (13 μm) **1^2+^** at 1 mm DPPC and 10 μm EY^2−^ overall ratio of **1^2+^**/EY^2−^ is 1:0.8 (n/n) upon irradiation with 375 nm LED light. Inset: Temporal evolution of the absorbance at 544 nm. b) Thermochemistry of energy transfer from photo excited **1^2+^** to EY^2−^ followed by electron transfer from excited state EY^2−^ to the water‐soluble electron acceptor EDTA^4−^.

To test the reactivity of the energy transferred on EY^2−^ for further redox reactions, DPPC:**1^2+^**:EY^2−^ liposomes (1000:13:10 n/n/n at 1 mm DPPC) were irradiated at 375 nm (0.5 mW) in the presence of an isotonic buffer containing 83 mm EDTA^4−^ at pH 7.8. During irradiation the absorption band at 544 nm characteristic for EY^2−^ vanished with a rate constant of 18 min^−1^, while simultaneously the absorption band of **1^2+^** was shifted from 351 nm to 354 nm. (Figure 3 a). Based on the excited state energies and redox potentials of all membrane‐embedded components or their reference compound (Table 2) the reaction sequence shown in Scheme 1 and Figure 3 is proposed. Upon photoexcitation of **1^2+^**, energy transfer (ET) takes place from an excited state of **1^2+^** to EY^2−^. This step has an overall driving force of 1.3 eV, either from the S_1_ state of **1^2+^** at 3.2 eV to the S_1_ state of EY^2−^ (2.3 eV) followed by intersystem crossing to the T_1_ state of EY^2−^ at 1.9 eV,^[40]^ or via inter system crossing of **1^2+^** to the T_1_ state at 2.3 eV,^[49]^ followed by triplet‐triplet energy transfer to the triplet excited state of EY^2−^ at 1.9 eV.^[40]^ From its T_1_ state EY^2−^ accepts an electron and two protons from the electron donor EDTA^4−^ with a driving force Δ*G*_eT_=−0.2 eV, providing the almost colorless EYH_2_^2−^.^[50]^

**Table 2:** Excited state energy (*E*_0‐0_) and electrochemical properties of the investigated compounds.

	*E*_0‐0_ [eV]	*E*_ox_ (V vs. SCE)	*E*_red_ (V vs. SCE)	Ref.
**1**(PF_6_)_2_ in MeCN		1.15	−2.13 (irrev.)	this study
**2** in MeCN *2 in MeCN	3.2 (S_1_‐state)^[49]^ ≈2.3 (T_1_‐state)^[49]^	1.17 2.03	−2.72 0.48	this study and [49]
EY^2−^ *EY^2−^	1.9 (T_1_‐state)	0.78 −1.1	−1.06 0.8	[40]
EDTA^4−^ in water		0.6		[51]

The slow electron transfer kinetics on the minute time scale can be explained by the strong association of the relatively hydrophobic EY^2−^ dyes to the membrane, as supported by the strong association constant with **1** ^2+^ and the close contact observed in molecular dynamics simulation (Supporting Info page S22–S23). By contrast, the strongly charged and poorly hydrophobic species EDTA^4−^ is anticipated to remain in the aqueous phase. Still, the positive charge of the antenna **1^2+^** might play a role in attracting the anionic EDTA^4−^ electron donor near the membrane‐water interface, thereby promoting electron transfer from the excited state of EY^2−^. As an alternative, it may also be possible that in DPPC:**1^2+^**:EY^2−^ liposomes EY^2−^ diffuses temporarily away from the membrane into the solution, to absorb photons by itself and directly photoreact with the sacrificial donor EDTA^4−^ in the aqueous phase, before stochastically coming back to the membrane.

To investigate if the observed photoreduction may have occurred via direct photoexcitation of EY^2−^ by the 375 nm exciting light (0.1×10^4^ m ^−1^ cm^−1^) and subsequent photoreduction by EDTA^4−^, we realized two control experiments. First, a strongly membrane‐bound eosin Y dye C16EY^−^ was prepared by covalent functionalization of the acid side group with a long (C16) aliphatic chain (Scheme 2). DPPC liposomes doped with 1 mol % of C16EY^−^ showed an absorption band similar to EY^2−^ at pH 7.8 in water, but red‐shifted to 545 nm. This is in line with the integration of the eosin dye into a hydrophobic environment such as a lipid bilayer.[^39^, ^42^] Irradiating DPPC:C16EY^−^ liposomes with neither 375 nm nor 530 nm light in the presence of EDTA^4−^ (42 mm) did not yield any spectroscopic changes. Therefore, no light‐induced electron transfer occurred between the strongly membrane bound excited state of C16EY^−^ and EDTA^4−^ in the aqueous phase. Secondly, free eosin EY^2−^ (6.7 μm) was quickly photoreduced in the presence of EDTA^4−^ (42 mm) in homogeneous, liposome‐free buffer at pH 7.8 upon irradiation with 375 nm LED light (0.5 mW), as seen by the disappearance of the absorption band at 517 nm with a rate constant of 1.15±0.1 min^−1^. The evolution of the spectra is shown in Figure S19. This photoreaction rate is significantly faster than that observed with DPPC:C16EY^−^ liposomes and DPPC:**1^2+^**:EY^2−^ liposomes, which is most probably due to a combination of several effects. First, in absence of **1** ^2+^ there is no filter effect by this strongly UV‐absorbing molecule, so all available light is absorbed by EY^2−^ and can lead to excited state formation. For DPPC:**1^2+^**:EY^2−^ liposomes, **1^2+^** absorbs most light, preventing direct absorption by EY^2−^. Second, diffusion rates are higher in homogeneous solution than with molecules embedded in membranes, which may improve electron transfer rate in liposome‐free conditions. Finally, in DPPC:**1^2+^**:EY^2−^ liposomes the strong association of EY^2−^ to **1^2+^** leads to a very low bulk concentration of EY^2−^ in the water phase, which slows down direct electron transfer from the excited states of EY^2−^, to EDTA^4−^.

## Conclusions

Overall, our experimental and theoretical data are consistent with the following picture. First, the transmembrane oligofluorene **1^2+^** is acting as a light‐harvesting chromophore that self‐assembles perpendicular to the membrane, and transfers photochemical energy to EY^2−^ within a membrane‐embedded supramolecular complex. We propose that following energy transfer, the triplet excited state of EY^2−^ is reduced at the membrane‐water interface by the reductant EDTA^4−^, to a colorless form. To the best of our knowledge, the combination of light absorption, energy transfer, and electron transfer using a transmembrane chromophore represents an unprecedented functional mimic of PS I using simple organic chromophores.

## Experimental Section

Experimental details including synthetic procedures can be found in the Supporting Information.

Deposition Number 1970033 contains the supplementary crystallographic data for the structure of the brominated intermediate obtained during the synthesis of **1^2+^**. These data are provided free of charge by the joint Cambridge Crystallographic Data Centre and Fachinformationszentrum Karlsruhe Access Structures service www.ccdc.cam.ac.uk/structures.

## Conflict of interest

The authors declare no conflict of interest.

## Supporting information

As a service to our authors and readers, this journal provides supporting information supplied by the authors. Such materials are peer reviewed and may be re‐organized for online delivery, but are not copy‐edited or typeset. Technical support issues arising from supporting information (other than missing files) should be addressed to the authors.

Supplementary

Supplementary
