# Supplementary material for: Mimicking Photosystem I with a Transmembrane Light Harvester and Energy Transfer‐Induced Photoreduction in Phospholipid Bilayers
Source: Chemistry. 2020 Dec 21;27(9):3013–8. doi: 10.1002/chem.202003391 (PMC7898337; doi:10.1002/chem.202003391)
Supplement: Supplementary file 1 — Supplementary [file CHEM-27-3013-s001.pdf]

Chemistry–A European Journal

Supporting Information

Mimicking Photosystem I with a Transmembrane Light Harvester and Energy Transfer-Induced Photoreduction in Phospholipid Bilayers

Andrea Pannwitz,*[a] Holden Saaring,[a] Nataliia Beztsinna,[a] Xinmeng Li,[a] Maxime A. Siegler,[b] and Sylvestre Bonnet*[a]

Author Contributions

A.P. Conceptualization: Lead; Data curation: Lead; Funding acquisition: Lead; Investigation: Lead; Methodology: Lead; Supervision: Equal; Writing - Original Draft: Lead; Writing - Review & Editing: Lead
H.S. Data curation: Supporting; Investigation: Supporting; Methodology: Supporting; Writing - Review & Editing: Supporting
N.B. Data curation: Supporting; Methodology: Supporting; Software: Supporting; Visualization: Supporting; Writing - Review & Editing: Supporting
M.S. Data curation: Supporting; Investigation: Supporting; Writing - Review & Editing: Supporting
S.B. Funding acquisition: Supporting; Resources: Lead; Supervision: Equal; Writing - Original Draft: Equal; Writing - Review & Editing: Equal.

Contents

1 Abbreviations 3

2 Methods 3

3 Synthesis 6

Synthesis route and general conditions 6

9,9-dimethylfluorene trimer (2, fl_3)[20] 7

Dibromo-9,9-dimethylfluorene trimer (Br-fl_3-Br) 8

(4-(dimethylamino)phenyl)boronic acid[21] 8

Bis(4-(dimethylamino)phenyl)-9,9-dimethylfluorene trimer (NMe_2-ph-fl_3-ph-NMe_2)[22] 9

$1(PF_6)_2$ 10

Eosin Y hexadecyl ester (C16EYH) [23] 11

4 NMR Spectra 12

$1(PF_6)_2$ 12

2 (fl_3) 13

5 Single Crystal X-ray Crystallography 14

6 Molecular Modeling for Figure 1a 17

7 Molecular Dynamics Simulations with GROMACS 19

8 Optical spectra 24

9 TD-DFT 25

10 Liposome Stability Tests 33

9 Stern-Volmer quenching experiment 36

10 Confocal microscopy 37

11 Eosin Y species and EDTA 40

12 References 41

1 Abbreviations

sat.	saturated
aq.	aqueous
rt	room temperature
DMPC	1,2-dimyristoyl-sn-glycero-3-phosphocholine
DPPC	1,2-dipalmitoyl-sn-glycero-3-phosphocholine
NaDSPE-PEG2K	sodium 1,2-dimyristoyl-sn-glycero-3-phosphoethanolamine-N-[methoxy(poly-ethylene glycol)-2000]
THF	tetrahydrofuran
dmso	dimethyl sulfoxide
MeOH	methanol
NPT	constant amount of substance, pressure and temperature in molecular dynamic simulations
NVT	constant amount of substance, volume and temperature in molecular dynamic simulations

2 Methods

Nuclear Magnetic Resonance Spectroscopy (NMR)

A Bruker AV300/1 FT-NMR spectrometer was used to record ^{1}H-NMR, ^{13}C-NMR, as well as COSY, HSQC and HMBC 2D spectra. Mestre Nova was used for the evaluation of the spectra.

Mass Spectrometry

The mass spectra of the compounds were obtained using a ThermoFischer Scientific MSQ Plus electrospray ionization mass spectrometer with a 17 – 2000 m/z detection range and a resolution of approximately 0.5 m/z.

Column Chromatography

Chromatographic silica columns were used for separating the components of the reaction mixtures, with a particle size of 40 – 63 µm and a surface area of 450 – 550 m^2/g. The pore volume of the particles was 0.75 – 0.85 cm^3/g. The silica powder was obtained from Screening Devices b.v.. Dry loading was carried out by adsorbing the mixture onto either celite or silica powder and solvent removal *in vacuo*, followed by deposition of the adsorbate on top of the column. The celite was obtained from Sigma-Aldrich.

Thin Layer Chromatography

Thin layer chromatography (TLC) was used as a first-hand method to test for the reaction mixtures' compositions and the products' purities. The TLC plates, composed of fluorescent silica matrix with a pore volume of 0.75 cm^3/g and a thickness of 0.2 mm, were supported on an aluminum sheet backing. The TLC plates were purchased from Supelco Analytical/Sigma Aldrich.

Elemental Analysis

Elemental analysis was performed by Mikroanalytisches Laboratorium Kolbe in Oberhausen, Germany. The elemental content of the molecules was reported as the elements' mass fraction percentage.

UV-vis Spectroscopy

Electronic absorption spectra were recorded on a Horiba Scientific Aqualog® or on an Agilent Cary 50 Scan UV-vis spectrophotometer equipped with a single cell Peltier temperature controller.

Luminescence Spectroscopy

Luminescence were recorded on a Horiba Scientific Aqualog® spectrophotometer equipped with a 150 W xenon lamp, excitation range of 230 – 620 nm, emission range 250 – 620 nm with 3.5 mL 10x10 mm quartz cuvettes with four polished sides were used.

Time resolved Luminescence

Luminescence lifetimes were recorded with a LifeSpec-II from Edinbourgh Scientific using a 375 nm source and recording the signal at 450 nm.

Dynamic Light Scattering (DLS)

The size distribution of the hydrodynamic diameter (Z_{Avg}) and the polydispersity index of liposomes was measured at 20 °C with a Zetasizer Nano-S from Malvern operating at 632.8 nm with a scattering angle of 173°.

Confocal Microscopy

Microscopy images were acquired on a Leica SP8 confocal microscope, equipped with 405 nm and 522 nm laser as excitation source. Images were processed with the Fiji version of ImageJ2.

Vesicle Preparation

The lipids 1,2-dimyristoyl-sn-glycero-3-phosphocholine (DMPC) and 1,2-dipalmitoyl-sn-glycero-3-phosphocholine (DPPC) were commercially available, as well as the additive N-(carbonyl-methoxypolyethylene glycol-2000)-1,2-distearoyl-sn-glycero-3-phosphoethanol-amine (NaDSPE-PEG2K). At room temperature, stock solutions of DMPC (5.0 mM) or DPPC (5.0 mM) and, if applicable NaDSPE-PEG2K (1.0 mM), in $CHCl_3$ were combined in the desired ratio in a pressure resistant glass tube. Additives in either $CHCl_3$ or methanol could be added to this mixture. The organic solvents were carefully evaporated, depositing a lipid film

on the inside of the tube wall. The film was dried under high vacuum for at least one hour. Rehydration of the lipid films occurred with the appropriate buffer via three successive cycles of freeze-thawing, using liquid N_2 and a water bath at 20 K above the respective transition temperature (T_m) of the lipid. This procedure yielded giant vesicles. From this mixture liposomes were prepared by extruding 11× with an Avanti Polar Lipids mini-extruder at 20 K above T_m using 200 nm cellulose membrane filters. The obtained clear solution contained monodisperse liposomes with a typical Z_{Avg}-diameter of around 150 nm according to DLS. These liposomes remained stable for more than one week at room temperature according to DLS.

3 Synthesis

Synthesis route and general conditions

All reagents were commercially available. Dry and degassed solvents were purified by a solvent purification system. (9,9-Dimethyl-9H-fluoren-2-yl)boronic acid, and 2,7-dibromo-9,9-dimethylfluorene were purchased from Fluorochem. The syntheses of the **2** (fl_3)[20] and (4-(di-methylamino)phenyl)boronic acid[21] are adapted from previously reported procedures on the same or other molecules.

All reactions were carried out under inert atmosphere, using standard Schlenk-techniques and N_2 gas. Room temperature was usually between 20 °C and 25 °C.

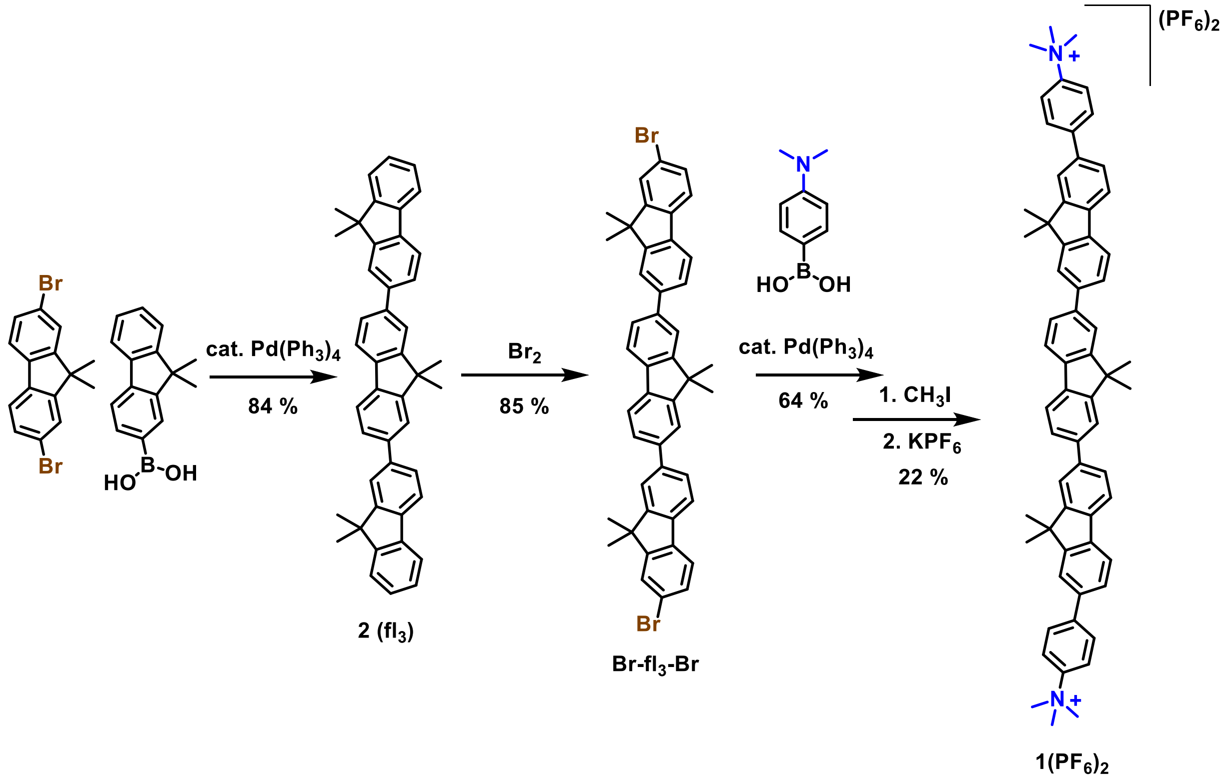

Scheme S1. Synthesis of **1**$(PF_6)_2$ and **2** (fl_3).

The synthesis of **1**$(PF_6)_2$ was performed in four consecutive steps involving two twofold Suzuki couplings. After the first Suzuki coupling between the 2,7-dibromo-9,9-dimethyl fluorene monomer and the single boronic acid of 9,9-dimethyl fluorene yielded the unsubstituted fluorene trimer **2** (fl_3) was obtained. Bromination with bromine occurred in the dark and at the two ends of the rod-like structure with high selectivity. Single crystals of this compound (**Br-fl3-Br**) were obtained and revealed that overhalogenation occurred with approximately 15 % of the substrate. The crystal structure is discussed *vide infra*, and the side

products were separated via the purifications in the follow-up steps. Reacting the halogenated fluorene trimer with the 4-boronic acid of dimethylaniline followed by methylation and counter ion exchange yielding the desired molecule as PF_6 salt. All intermediates were characterized, with ESI-MS if possible, and ^{1}H-and ^{13}C-NMR spectroscopy, including 2D spectra for assignment of the peaks for most of the spectra.

9,9-dimethylfluorene trimer (2, fl_3)[20]

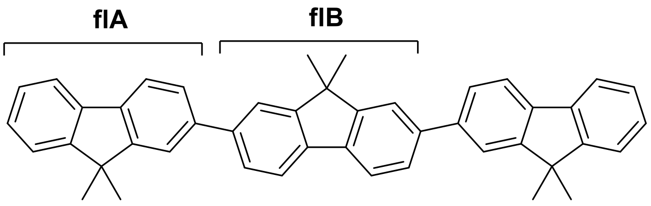

fl_3

A degassed mixture of 2,7-dibromo-9,9-dimethylfluorene (400 mg, 1.14 mmol, 1.00 eq.), (9,9-dimethyl-9H-fluoren-2-yl)boronic acid (650 mg, 2.73 mmol, 2.40 eq.), Na_2CO_3 (7.23 mg, 6.82 mmol, 6.00 eq.), and $Pd(PPh_3)_4$ (117 mg, 101 µmol, 0.09 eq.) in THF (12 mL) and water (3.4 mL) were stirred at reflux for 14 h. After cooling to room temperature, water (50 mL) was added, and the mixture was extracted with CH_2Cl_2 (3 × 50 mL). The combined organic phases were dried over $MgSO_4$ and the solvent was removed at reduced pressure. Purification by column chromatography (SiO_2, pentane → pentane/CH_2Cl_2 200:20 → pentane/CH_2Cl_2 200:50 → CH_2Cl_2) afforded the target compound as a white solid (550 mg, 952 µmol, 84 %) which was suitable for the next synthesis steps.

A second column chromatography (SiO_2, pentane → pentane/CH_2Cl_2 10:1 → pentane/CH_2Cl_2 20:5 → CH_2Cl_2) afforded the target compound, suitable for spectroscopy.

***R*f** = 0.29 (pentane/DCM 20:4).

UV-vis ($CHCl_3$): λ_{max} = 358 nm (6.8 · 10^4 L mol^{-1} cm^{-1}).

Luminescence ($CHCl_3$): λ_{em} = 393 nm, 414 nm.

^{1}H NMR (300 MHz, $CDCl_3$): δ (ppm) = 7.88 – 7.63 (m, 14H), 7.51 – 7.44 (m, 2H), 7.42 – 7.30 (m, flA-4H), 1.66 (s, 6H, flB-CH_3), 1.58 (s, 12H, flA-CH_3).

^{13}C-APT NMR (75 MHz, $CDCl_3$): δ (ppm) = 154.76 (C^q), 154.46 (C^q), 154.07 (C^q), 140.99 (C^q), 140.97 (C^q), 139.06 (C^q), 138.56 (C^q), 138.23 (C^q), 127.39 (CH), 127.20 (CH), 126.55 (CH), 126.45 (CH), 122.78 (CH), 121.60 (CH), 121.57 (CH), 120.52 (CH), 120.46 (CH), 120.22 (CH), 47.31(1C, flB-$\underline{C}^q$-CH_3), 47.16 (2C, flA-$\underline{C}^q$-CH_3), 27.57 (4C, flB-CH_3), 27.44 (4C, flA-CH_3).

Dibromo-9,9-dimethylfluorene trimer (Br-fl3-Br)

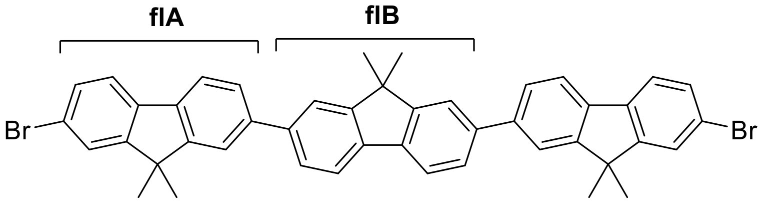

Br-fl3-Br

A solution of compound **2** (fl3) (300 mg, 518 µmol, 1.00 eq.) in $CHCl_3$ (10 mL) was degassed and cooled to 0 °C. In the dark, a grain of I_2 (2.6 mg. 10 µmol, 0.02 eq.) was added, followed by dropwise addition of Br_2 (60 µL, 182 mg, 1.14 mmol, 2.20 eq.). The mixture was then stirred at room temperature for 44 h. Addition of sat. aq. $Na_2S_2O_3$ (10 mL) and water (10 mL) was followed by phase separation and extraction of the aq. layer with CH_2Cl_2. The combined organic phases were dried over $MgSO_4$, which was filtered off. The organic solvent was removed, and the crude product, dissolved in CH_2Cl_2, was filtered over a plug of SiO_2 and collected with CH_2Cl_2. The solvent was removed under reduced pressure and the product was obtained as a white solid (366 mg, 497 µmol, 96 %).

$\boldsymbol{R}_{\mathbf{f}}$ = 0.85 (pentane/DCM 1:1).

^{1}H NMR (300 MHz, $CDCl_3$): δ (ppm) = 7.91 – 7.56 (m, 16H), 7.49 (dd, *J* = 8.1, 1.8 Hz, 2H), 1.64 (s, 6H, flB-CH_3), 1.57 (s, 12H, flA-CH_3).

^{13}C-APT NMR (75 MHz, $CDCl_3$): δ (ppm) = 156.10 (C^q), 154.78 (C^q), 154.11 (C^q), 141.42 (C^q), 140.82 (C^q), 138.31 (C^q), 138.06 (C^q), 137.47 (C^q), 130.34 (C^q), 126.67 (C^q), 126.57 (CH), 126.34 (CH), 121.58 (CH), 121.20 (flA-C^q-Br), 120.58 (CH), 120.55 (CH), 47.41 (2C, flA-$\underline{C^q}$-CH_3), 47.30 (1C, flB-$\underline{C^q}$-CH_3), 27.54 (2C, flB-CH_3), 27.29 (4C, flA-CH_3).

(4-(dimethylamino)phenyl)boronic acid[21]

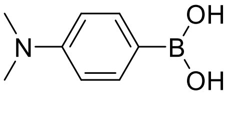

To 4-bromo-N,N-dimethylaniline (5.00 g, 25.0 mmol, 1.00 eq.) in anhydrous THF (40 mL) at -78°C was added n-butyl lithium in hexane (2.5 M, 11.0 mL, 1.76 g, 27.5 mmol, 1.10 eq.) dropwise via a syringe. After stirring at -78 °C for 1 h, triethyl borate (4.25 mL, 3.65 g, 25.0 mmol, 1.00 eq.) was added dropwise and stirring was continued at -78 °C for another hour. The mixture was allowed to warm up to room temperature and stirring continued for 4 days. To the

mixtures was added aqueous HCl (1M, 50 mL) and stirring continued for 2 h. Neutralization occurred with sat. aq. Na_2CO_3 resulting in a white precipitate that was extracted into the organic phase with CH_2Cl_2 (3×150 mL). The combined organic phases were dried over $MgSO_4$ and solvent evaporation yielded the desired product as a white solid (3.72 g, 22.6 mmol, 90 %).

Recrystallization from cold CH_2Cl_2 did not increase the purity of the product, which was suitable for the next synthesis step. The ^{1}H NMR spectra were recorded in $CDCl_3$ and in reasonable agreement with the reported ^{1}H NMR spectra in dmso-d_6.[21]

^{1}H NMR (300 MHz, $CDCl_3$): δ (ppm) = 8.13 – 8.04 (m, 2H, H-2, H-6), 6.78 (d, *J* = 8.7 Hz, 2H, H-3, H-5), 3.05 (s, 6H, CH_3), 2.94 (s, 2H, OH).

^{13}C NMR (75 MHz, $CDCl_3$): δ (ppm) = 153.19 (C-N(CH_3)$_2$), 137.20 (C-2, C-6), 111.35 (C-3, C-5), 95.78 (C-B(OH)$_2$), 40.30 (CH_3).

Bis(4-(dimethylamino)phenyl)-9,9-dimethylfluorene trimer (NMe_2-ph-fl$_3$-ph-NMe_2)[22]

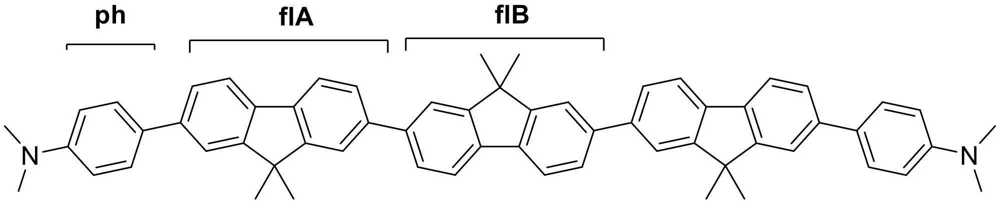

NMe_2-ph-fl$_3$-ph-NMe_2

A mixture of dibromo-9,9-dimethylfluorene trimer (**Br-fl$_3$-Br**) (100 mg, 136 µmol, 1.00 eq.), 2,5-(4-(dimethylamino)phenyl)boronic acid (67.2 mg, 407 µmol, 3.00 eq.), Cs_2CO_3 (157 mg, 815 µmol, 6.00 eq.) and Pd(PPh_3)$_4$ (31.4 mg, 27.2 µmol, 0.20 eq.) in dry and degassed toluene (10 mL) were heated at reflux for 4 days. After cooling to room temperature, sat. aq. Na_2CO_3 (20 mL) was added and the mixture was extracted with a mixture of CH_2Cl_2, $CHCl_3$ and diethyl ether. The combined organic phases were washed with sat. aq. Na_2CO_3, brine and water. After removing the organic solvent the crude product was purified with column chromatography (SiO_2, pentane/toluene/NEt_3 2:1:(1%) → toluene/NEt_3 1:(1%) → toluene/MeOH/NEt_3 10:1:(1%)) yielding the desired product as a yellow solid (45.0 mg, 53 µmol, 40 %).

***R*f** = 0.3 (toluene).

^{1}H NMR (300 MHz, $CDCl_3$): δ (ppm) = 7.91 – 7.48 (m, 22H), 6.86 (s, 4H, ph-CH), 3.04 (s, 12H, N(CH_3)$_2$), 1.66 (s, 6H, flB-CH_3), 1.62 (s, 12H, flA-CH_3).

1$(PF_6)_2$

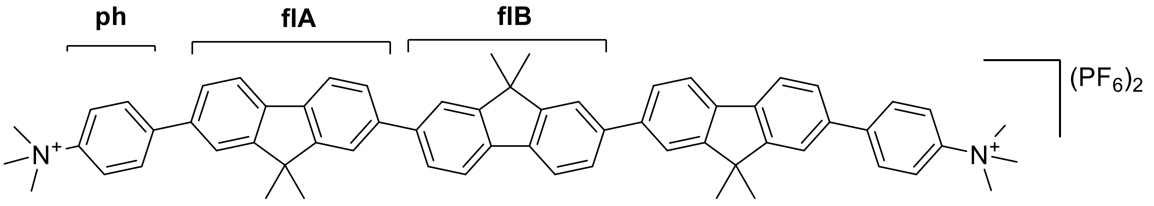

1$(PF_6)_2$

A mixture of **NMe_2-ph-fl_3-ph-NMe_2** (30 mg, 36.7 µmol, 1.00 eq.) and methyl iodide (1.00 mL, 2.28 g, 16.1 mmol, 438 eq.) in acetonitrile (5 mL), in a pressure tube were heated at reflux overnight. After cooling to room temperature 1 M aqueous KOH was added and stirring was continued for 30 min to quench the excess methyl iodide. Sat. aq. KPF_6 (3 mL) was added, the organic solvent was removed in vacuo, and the crude product was filtered and washed with water. Column chromatography (SiO_2, CH_2Cl_2 → acetone → acetone/water/sat. aq. KPF_6 100:10:2 → acetone/sat. aq. KPF_6 6:1). The organic solvent was removed in vacuo and the precipitate was filtered and washed with water yielding the desired product as off-white solid (12.0 mg, 10.6 µmol, 29 %).

R_f = 0.75 (acetone/sat. aq. KPF_6 6:1).

^{1}H NMR (300 MHz, Acetone-*d_6*) δ (ppm) = 8.25 – 8.16 (m, 4H, ph-CH meta to $N(CH_3)_3$), 8.16 – 8.04 (m, 4H, ph-CH ortho to $N(CH_3)_3$), 8.04 – 7.93 (m, 12H, fl_3), 7.86 – 7.73 (m, 6H, fl_3), 3.97 (s, 18H, $N(CH_3)_3$), 1.67 (s, 6H, flB-CH_3), 1.66 (s, 12H, flA-CH_3).

^{13}C NMR (75 MHz, Acetone-d_6) δ (ppm) = 155.78 (fl-$\underline{C}^q$-C^q-$(CH_3)_2$), 155.84 (fl-$\underline{C}^q$-C^q-$(CH_3)_2$), 155.89 (fl-$\underline{C}^q$-C^q-$(CH_3)_2$), 156.04 (fl-$\underline{C}^q$-C^q-$(CH_3)_2$), 155.69 (fl-$\underline{C}^q$-C^q-$(CH_3)_2$), 155.55 (fl-$\underline{C}^q$-C^q-$(CH_3)_2$), 147.15 (ph-$\underline{C}^q$-$N(CH_3)_3$), 129.46 (ph-$\underline{CH}$ ortho to $N(CH_3)_3$), 127.31 (fl-CH), 127.25 (fl-CH), 127.19 (fl-CH), 122.48 (fl-CH), 122.34 (fl-CH), 122.32 (fl-CH), 121.67 (fl-CH), 121.62 (ph-$\underline{CH}$ meta to $N(CH_3)_3$), 121.44 (fl-CH), 57.92 (N-CH_3), 47.99 (flB-$\underline{C}^q$-$(CH_3)_2$), 47.91 (flA-$\underline{C}^q$-$(CH_3)_2$), 27.49 (flA-CH_3), 27.38 (flB-CH_3).

ESI-MS: calcd. (m/z) for $C_{63}H_{62}N_{2}{}^{2+}$ $[M]^{2+}$: 423.2, found: 423.6.

UV-vis (MeOH): λ_{max} = 358 nm ($16 \cdot 10^4$ L mol^{-1} cm^{-1}).

Luminescence (MeOH): λ_{em} = 404 nm, 422 nm.

Anal. Calcd. for $C_{63}H_{62}F_{12}N_2P_2 \cdot 1\ H_2O$: C 65.51, H 5.58, N 2.43, found: C 65.60, H 5.44, N 2.42.

Eosin Y hexadecyl ester (C16EYH) [23]

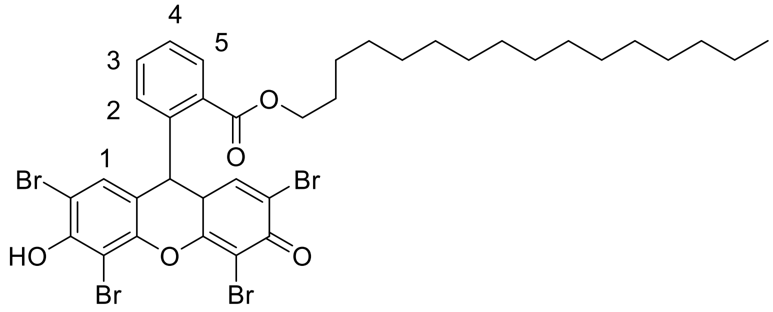

C16EYH

To a solution of eosin Y disodium salt (100 mg, 145 µmol, 1.0 eq.) in DMF (5 mL), 1-bromo-n-hexadecane (44.5 µL, 145 µmol, 1.0 eq.) was added. The mixture was stirred at 60 °C for 3 h. The mixture was cooled to room temperature. An equal volume of toluene (5 mL) was added to the flask, followed by vigorous stirring. The contents of the flask were transferred into a 50 mL separatory funnel along with 10 mL of 1 M HCl. After phase separation, the organic layer was washed with water (10 mL) and the solvent was removed *in vacuo*. Once finished, the mixture was cooled to room temperature. An equal volume of toluene (5 mL) was added to the flask, followed by a vigorous stirring. The contents of the flask were transferred into a 50 mL separatory funnel along with 10 mL of 1 M HCl. The layers were allowed to phase-separate. The organic phase was washed with water (2 × 10 mL) and the solvent was removed *in vacuo*. Column chromatography (SiO_2, DCM → acetone) yielded the product as the second of three fractions (R_f-s = 0.62; 0.27 and 0.16) as a waxy rose-red solid (55.68 mg, 63.8 µmol, 44%).

$\boldsymbol{R_f}$ = 0.27 (DCM/acetone 10:1).

^{1}H NMR (300 MHz, Methanol-*d*$_4$) δ (ppm) = 8.26 (dd, J = 7.2, 1.9 Hz, 1H, H-5), 7.81 (ddt, J = 7.3, 1.6 Hz, 2H, H-4, H-3), 7.42 (dd, J = 6.9, 1.9 Hz, 1H, H-2), 7.16 (s, 2H, H-1), 3.93 (t, J = 6.1 Hz, 2H, O$\underline{CH_2}$), 1.26 (s, 28H, 14xCH_2), 0.88 (t, J = 6.6 Hz, 3H, CH_3).

ESI-MS (CH_3CN): $[M+H]^+$ calcd. (m/z) for $C_{36}H_{41}Br_4O_5^+$: 873.3, found: 873.6.

UV-vis (MeOH): λ_{max} = 531 nm.

Luminescence (MeOH): λ_{em} = 556 nm.

Anal. Calcd. for $C_{36}H_{40}Br_4O_5 \cdot 0.75\ H_2O$: C 49.57, H 4.62; found: C 48.88, H 4.83.

4 NMR Spectra

$1(PF_6)_2$

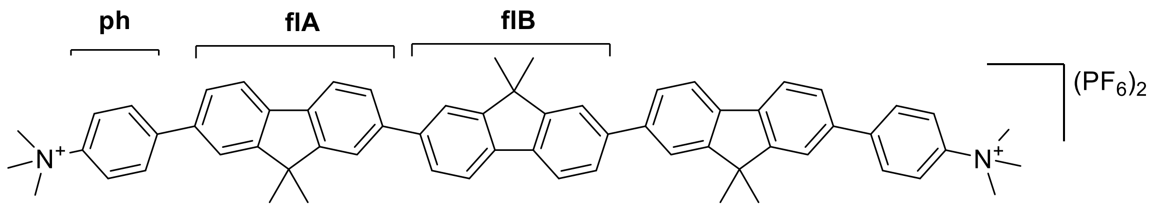

$1(PF_6)_2$

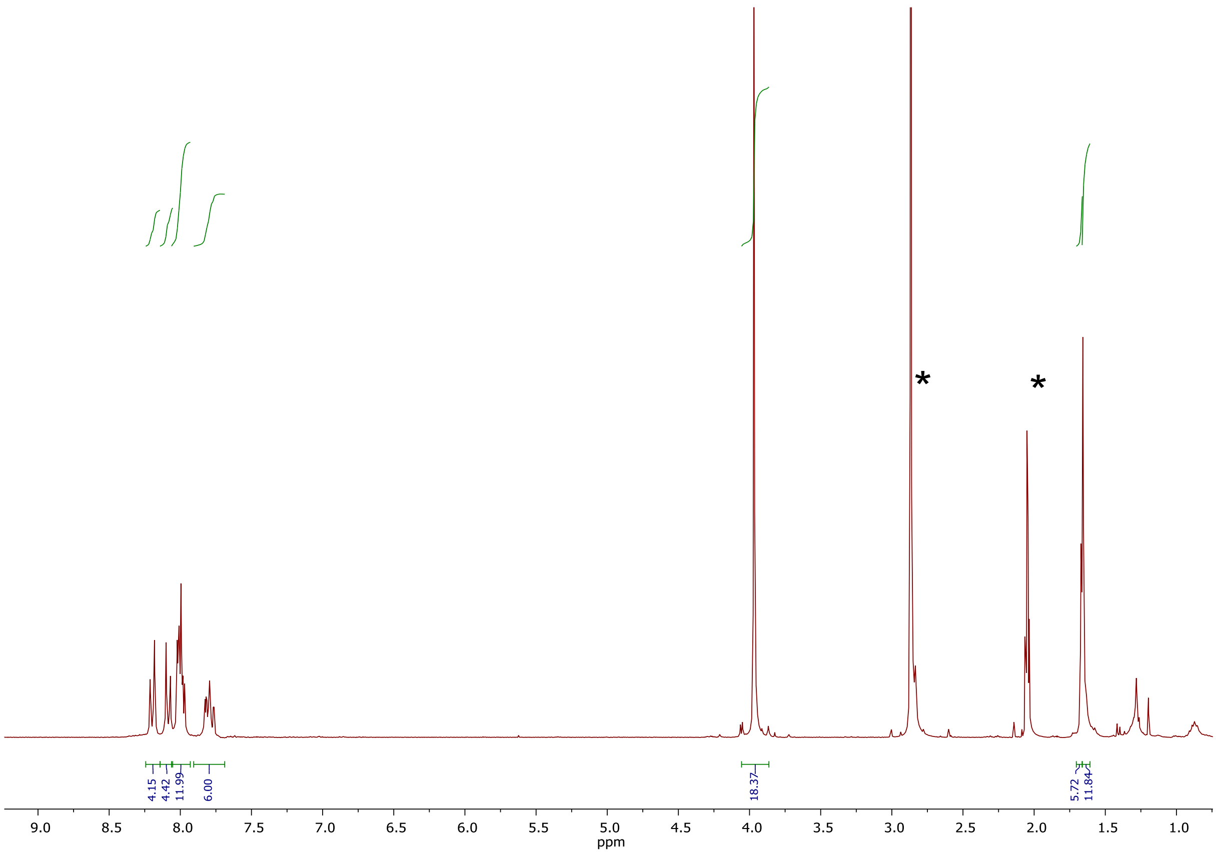

Figure S2. ^{1}H-NMR spectrum of **1**$(PF_6)_2$ in acetone-d_6. The asterisk (*) indicates the residual solvent peaks of water and acetone.

2 (fl3)

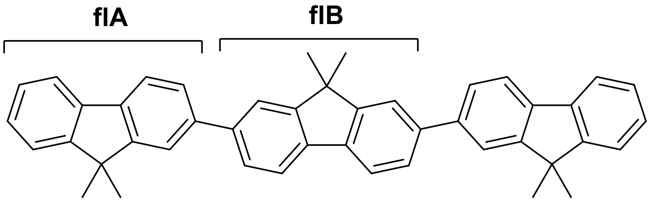

fl3

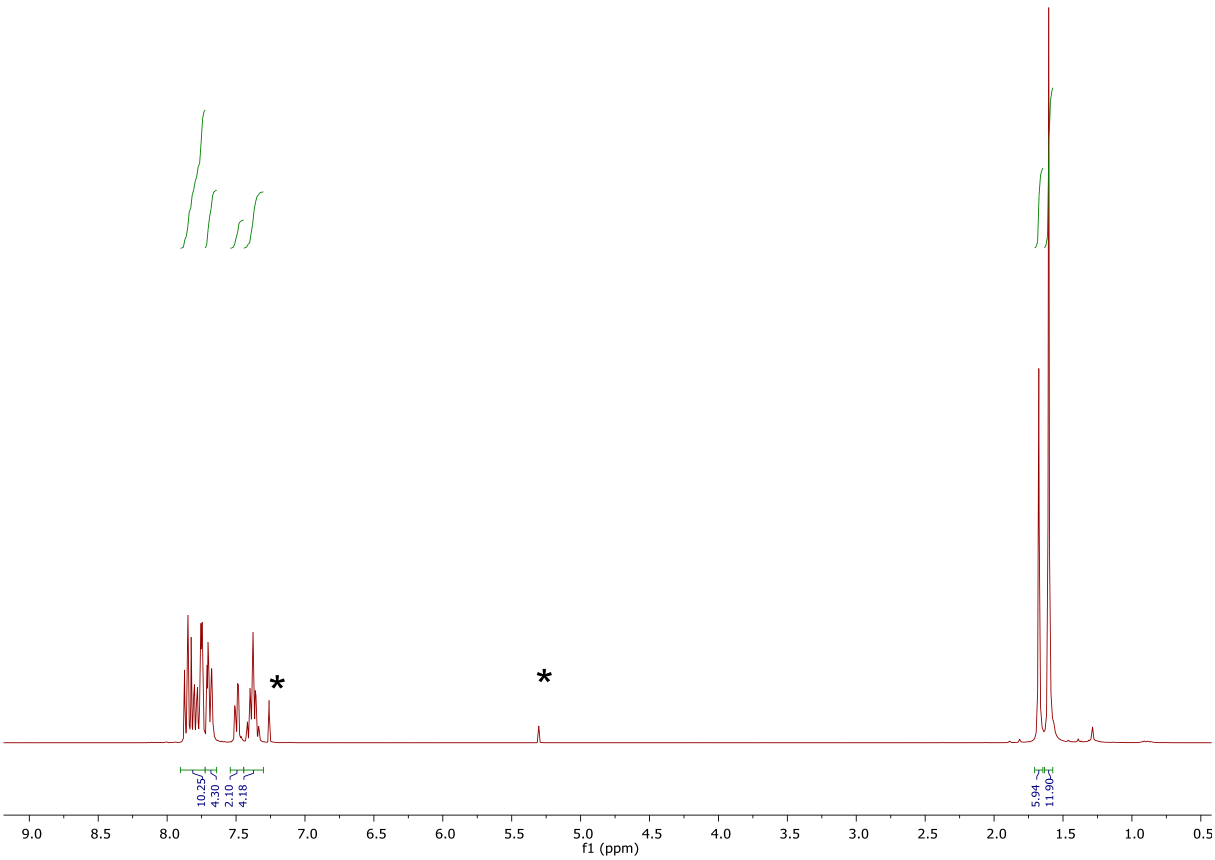

Figure S3. ^{1}H-NMR spectrum of **3** (fl3) in $CDCl_3$. The asterisk (*) indicates the residual solvent peaks of chloroform and CH_2Cl_2.

5 Single Crystal X-ray Crystallography

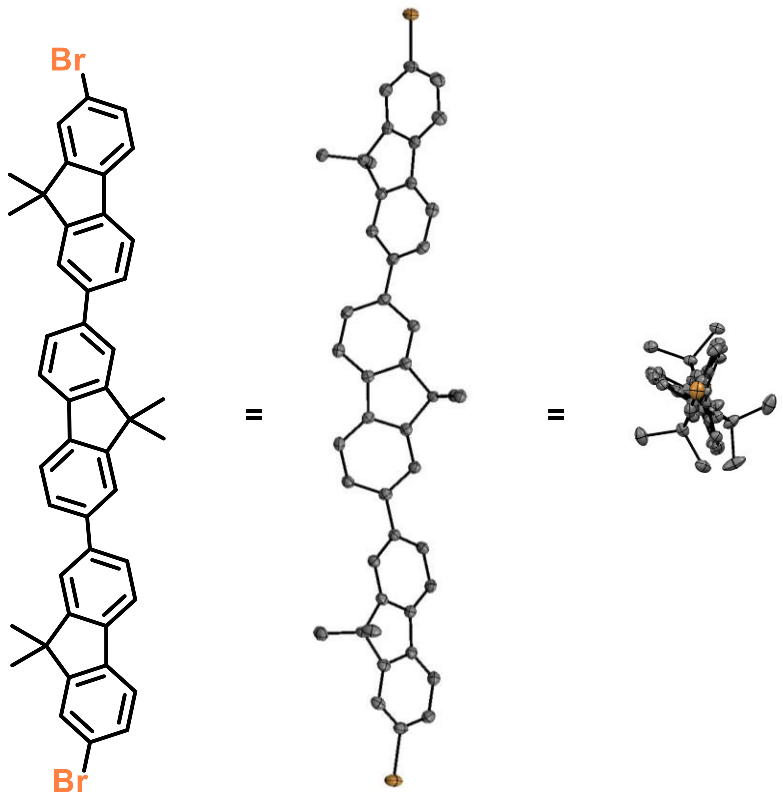

Br-fl$_3$-Br

Figure S4. Displacement ellipsoid plots (50% probability level) of the dibrominated 9,9-dimethy trifluorene at 110(2) K from two views showing the torsion of the single fluorene units with respect to each other. H atoms, lattice solvent molecules (dichloromethane) and disorder are omitted for clarity.

Colorless single crystals of the twofold brominated fluorene trimer were obtained by evaporation of the solvent dichloromethane at room temperature. The structure can be found under CCDC1970033.

All reflection intensities were measured at 110(2) K using a SuperNova diffractometer (equipped with Atlas detector) with Mo *K*α radiation (λ = 0.71073 Å) under the program CrysAlisPro (Version CrysAlisPro 1.171.39.29c, Rigaku OD, 2017). The same program was used to refine the cell dimensions and for data reduction. The structure was solved with the program SHELXS-2018/3 (Sheldrick, 2018) and was refined on F^2 with SHELXL-2018/3 (Sheldrick, 2018). Numerical absorption correction based on gaussian integration over a multifaceted crystal model was performed using CrysAlisPro. The temperature of the data

collection was controlled using the system Cryojet (manufactured by Oxford Instruments). The H atoms were placed at calculated positions using the instructions AFIX 23, AFIX 43 or AFIX 137 with isotropic displacement parameters having values 1.2 or 1.5 U_{eq} of the attached C atoms. The structure is partly disordered. The co-crystallized CH_2Cl_2 solvent molecules were disordered, as well as part of the brominated fluorene trimer, which exhibited some halogenation of the central carbons. The structure contains some amount of impurities near the sites C8, C23, C24 C27. Those impurities are thought to be some small amounts of Br (Br1' on C8) or I (I1', I2' and I3' on C23, C24 and C27, respectively), which are found less than 5% of the time. The atom Br2 is also slightly disordered over two orientations. The asymmetric unit also contains two disordered lattice CH_2Cl_2 solvent molecules. One molecule is disordered over two orientations, whereas the other molecule is found in four different orientations. All values of the occupancy factors can be retrieved from the .cif file.

Every single fluorene spans over (6.90 ± 0.03) Å (C2-C7-distance according to IUPAC for fluorene), and the fluorene trimer spans over 23.535(4) Å (terminal C-C distance), or 27.278(1) Å (Br-Br distance). The individual fluorene units propel around the core axis of the molecule in 39 ± 1° angles towards each other due to steric hindrance of hydrogens *ortho* to the connecting C-C bond.

Table S1. Single crystal X-ray crystallography data of Br-fl$_3$-Br

	**Br-fl$_3$-Br**
Crystal data	
Chemical formula	$C_{45}H_{35.85}Br_{2.05}I_{0.11}\cdot 2(CH_2Cl_2)$
M_r	923.57
Crystal system, space group	Monoclinic, $P2_1/c$
Temperature (K)	110
a, *b*, *c* (Å)	18.4565 (6), 14.6564 (4), 17.0146 (7)
β (°)	113.793 (4)
V (Å^3)	4211.4 (3)
Z	4
Radiation type	Mo *K*α
μ (mm^{-1})	2.34
Crystal size (mm)	0.31 × 0.17 × 0.13
Data collection	

Diffractometer	SuperNova, Dual, Cu at zero, Atlas
Absorption correction	Gaussian<br>*CrysAlis PRO* 1.171.39.29c (Rigaku Oxford Diffraction, 2017) Numerical absorption correction based on gaussian integration over a multifaceted crystal model Empirical absorption correction using spherical harmonics, implemented in SCALE3 ABSPACK scaling algorithm.
T_{min}, T_{max}	0.435, 1.000
No. of measured, independent and observed [$I > 2\sigma(I)$] reflections	50017, 9651, 7459
R_{int}	0.046
$(\sin \theta/\lambda)_{max}$ (Å^{-1})	0.650
Refinement	
$R[F^2 > 2\sigma(F^2)]$, $wR(F^2)$, S	0.046, 0.105, 1.07
No. of reflections	9651
No. of parameters	641
No. of restraints	245
H-atom treatment	H-atom parameters constrained
$\Delta\rho_{max}$, $\Delta\rho_{min}$ (e Å^{-3})	0.83, -0.56

6 Molecular Modeling for Figure 1a

Molecular Modeling was performed with YASARA, using the using the membrane simulation macro membranemd delivered with the program, which was originally developed by Elmar Krieger. The host lipid of the membrane was 1-palmitoyl, 2-oleoyl-choline. Equilibration for the modeling in Figure 1 was performed for 83 ns. Two complementing molecular modelings of $\mathbf{1}^{2+}$ in phospholipid bilayers with similar equilibration times yielded the same results considering the shape of $\mathbf{1}^{2+}$ and its position with respect to the phospholipid bilayer.

The source code of the simulation protocol and visualizations of the individual steps can be found at www.yasara.org/membranemd.

The simulation was set up automatically by first scanning the target molecule for exposed transmembrane helices (i.e. helices longer than 16 residues, with more than seven hydrophobic residues and more than three exposed ones (accessible side-chain surface area >30% of maximum)). The major axis vectors of these helices (i.e. the direction vectors of the least-squares lines through the Calpha atoms) were summed up to obtain the major axis of the target molecule, which was then oriented along the Y-axis, normally with respect to the plane of the membrane and the XZ-plane. The best shift of the membrane along this major axis was obtained by scanning the target molecule for the region with the largest number of exposed hydrophobic residues (see definition above) and a width of 28 Å (corresponding to the membrane core). Having placed an equilibrated membrane structure (consisting of phosphatidyl-ethanolamine molecules) at this location named 'MemCenterY', the system was enclosed in a simulation cell of size [X*Y*Z] Å, the target molecule was temporarily scaled by 0.9 along the XZ-axes, then strongly clashing membrane lipids were deleted (lipids with an atom closer than 0.75 Å to a target molecule atom). The temporary target molecule scaling, which was needed to avoid the deletion of too many lipids around the target molecule, was then slowly removed during a short simulation at 298K *in vacuo*: the target molecule (with all atoms kept fixed) was scaled by 1.02 along the XZ-axes every 200 femtoseconds, while the membrane was allowed to move, but rest ,rained to ideal geometry (by pulling lipid residues with an atom further than 21.5 Å away from MemCenterY back into the membrane, and by pushing phosphorus atoms closer than 14 Å to MemCenterY back outwards). The force field was AMBER14, with Lipid14/GAFF/AM1BCC parameters for non-standard residues. As soon as the target molecule had reached its original size again, the target molecule side-chain pKas were predicted,[1] protonation states assigned according to pH 7.4, and the simulation cell was filled with water, 0.9% NaCl and counter ions.[2] The main simulation was then run with PME and 8.0 Å cutoff for non-bonded real space

forces, a 4 fs time-step, constrained hydrogen atoms, and at constant pressure and temperature (NPT ensemble), as described in detail previously.[3] During the initial 250 picoseconds, the membrane was restrained to avoid distortions while the simulation cell adapted to the pressure exerted by the membrane (see above, additionally water molecules that got closer than 14 Å to MemCenterY were pushed outside).

7 Molecular Dynamics Simulations with GROMACS

We examined the stability of the $\mathbf{1}^{2+}$ in phospholipid bilayers. Here, we performed additional molecular dynamics (MD) simulations to (1) evaluate the capability and propensity of $\mathbf{1}^{2+}$ forming transmembrane configurations in bilayer lipids, and to (2) prove the existence of a stable association between EY^{2-} and $\mathbf{1}^{2+}$.

MD simulation Methods.

All simulations in this section were performed using the GROMACS 2018 software.[8] For the $\mathbf{1}^{2+}$, PF_6^- and EY^{2-}, the van der Waals and bonded parameters were taken from Amber14SB using the program acpype.[9,10] Their atomic point charge parameters were obtained by the RESP method based on the B3LYP/6-31G* level structure optimization and single point calculation using the Gaussian16 software.[11,12] The force field parameters of DMPC are taken from Lyubartsev's work,[13] which is compatible with Amber force fields. For water molecules, the tip3p model was used.[14]

All simulations were carried out using periodic boundary conditions. We sequentially performed energy minimization, NVT and NPT relaxation simulation, and NPT production simulation for each system. The targeting temperature and pressure are set to 300 K and 1 Bar. The velocity-rescale thermostat was used with a coupling constant of 0.1 ps.[15] The pressure coupling was applied using the Berendsen scheme, with a coupling constant of 1.0 ps.[16] In some semi-isotropic pressure coupling simulations, Parrinello-Rahman barostat with a coupling constant of 2.0 ps was also used.[17,18] Bond lengths were constrained using the LINCS algorithm, and simulations were run with a 2-fs time step. The van der Waals interactions were calculated using a cutoff of 1 nm. The electrostatic interactions were calculated using the PME method.[19]

Spontaneous aggregation simulation. We performed simulations that starting from initial configurations chosen as randomly distributed mixture of 128 DMPC lipids and one $1(PF_6)_2$ in a cubic water solvent box (7.0-nm edge length). Our simulation protocol is stated as follows. After energy minimization, 10 ps NVT (constant amount of substance, volume and temperature), and 40 ps NPT (constant amount of substance, pressure and temperature) simulations with isotropic pressure control are conducted with position constraints on the heavy atoms of $\mathbf{1}^{2+}$ molecules. A following 100 ns production NPT simulation allows the system to evolve freely under anisotropic pressure coupling. In the end, an extra 100 ns NPT simulation using semi-isotropic pressure coupling is performed to further equilibrate the formed

membrane. As the semi-isotropic pressure coupling in GROMACS is suitable for membranes that with their norm vectors along the z-axis, the membrane formed from the anisotropic simulation has to be oriented accordingly before the last 100 NPT simulation.

We observed six simulations with successfully formed bilayer membrane and **1**$^{2+}$ formed transmembrane configuration in four of them. The configuration of **1**$^{2+}$ was determined by visualization and by alignment angle analysis, upon the last snapshots of the simulations. Table S2 summarizes the simulated systems and the results of the alignment angle; Figure S5 lists all the snapshots. The alignment angle α is the angle between the direction vector of the **1**$^{2+}$ and the x-y plane, where the direction vector connects the two nitrogen atoms in the **1**$^{2+}$. For a small value, e.g. $\alpha < 20°$, the configuration is treated as parallel, and for a large α value, e.g. $\alpha > 60°$, the configuration is treated as perpendicular.

Table S2. List of six spontaneous aggregation simulations.

**simulation No.**	**number $1(PF_6)_2$**	**number DMPC**	**number water**	**alignment angle with respect to membrane surface** $\alpha(°)$	**Orientation with respect to membrane surface**
1	1	128	6131	62.7	transmembrane
2			6092	0.6	parallel
3			6129	64.9	transmembrane
4			6120	79.3	transmembrane
5			6186	84.1	transmembrane
6			6145	1.7	parallel

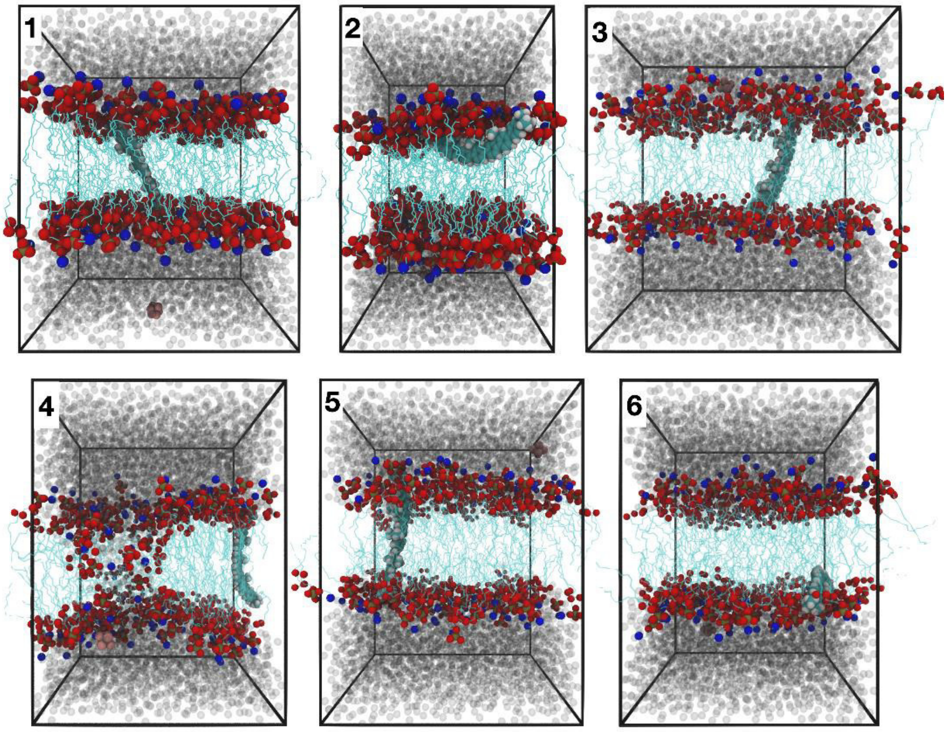

Figure S5. Visualization of the last snapshots of the simulations listed in Table S2. The number on the left corner corresponds to the simulation number in Table S2. All images are made by VMD software. Heavy atoms of the lipids and **1**$(PF_6)_2$ are shown by spheres (blue/red/cyan/white color for Nitrogen/Oxygen/Carbon/Hydrogen atoms). For ease of visualization, tails of lipids are shown by lines, and water molecules are shown by grey transparent spheres. In the aggregation process, there may exist a water pole in the membrane, as shown in the 4th simulation. In all other cases, such a water pole disappeared within the 200 ns NPT simulation time. To illustrate the evolution process, we showed the trajectory of the first simulation in the supporting information Movie1.mpg.

Binding free energy. After obtaining perpendicular and parallel configurations, it is also possible to compare their binding free energies between the $\mathbf{1}^{2+}$ and the membrane to estimate the preference over different transmembrane configurations. As for a quick estimation, we adopted the molecular mechanics Poisson-Boltzmann surface area (MM-PBSA) method[4] to calculate the binding free energy ΔG_{bind} using the g_mmpbsa software.[5] In particular, the SASA-only model is adopted, see more details about the parameters are given in the pbsa.mdp file in the g_mmpbsa official webpage: https://rashmikumari.github.io/g_mmpbsa/single_protein_ligand_binding_energy.html. Such

binding energy calculation has also been used in a recent study of peptide/membrane complexes.[6]

In particular, we focused on the 5th and 6th simulations and collected snapshots every one ns from the last ten ns trajectories to compute the binding free energy ΔG_{bind}. The averaged ΔG_{bind} for the perpendicular (5th) and parallel (6th) cases are -165.5 kJ/mol and -22.4 kJ/mol, respectively, which further confirms a propensity for the formation of transmembrane configuration. We note these values only serve for comparison, and more accurate methods may be needed to improve the accuracy,[7] which is beyond the scope of our current work.

Association between EY^{2-} and $\mathbf{1}^{2+}$. As a small separation distance between the donor and the acceptor will promote their energy transfer, we check the possibility of forming a stable close contacted EY^{2-}/ $\mathbf{1}^{2+}$ complex.

The initial configuration is of box size 6.29*6.33*6.36 nm^3, which contains one $\mathbf{1}^{2+}$, one EY^{2-}, two PF_6^-, two Na^+ ions, 125 DMPC, and 3815 water molecules. DMPC molecules formed a membrane in the center of the box with the norm direction along the z-axis. The $\mathbf{1}^{2+}$ molecule was aligned along the z-axis and embedded in the center of the membrane. To save computational time, the EY^{2-} molecule was put in the water/lipids interface and near the $\mathbf{1}^{2+}$ molecule. After energy minimization, a 200 ps NVT and a 500 ps NPT (semi-isotropic pressure coupling) were conducted with position constraints on all the heavy atoms of $\mathbf{1}^{2+}$, EY^{2-} and $PF6^-$ molecules. A following 30 ns production NPT simulation allowed the system to evolve freely under semi-isotropic pressure coupling.

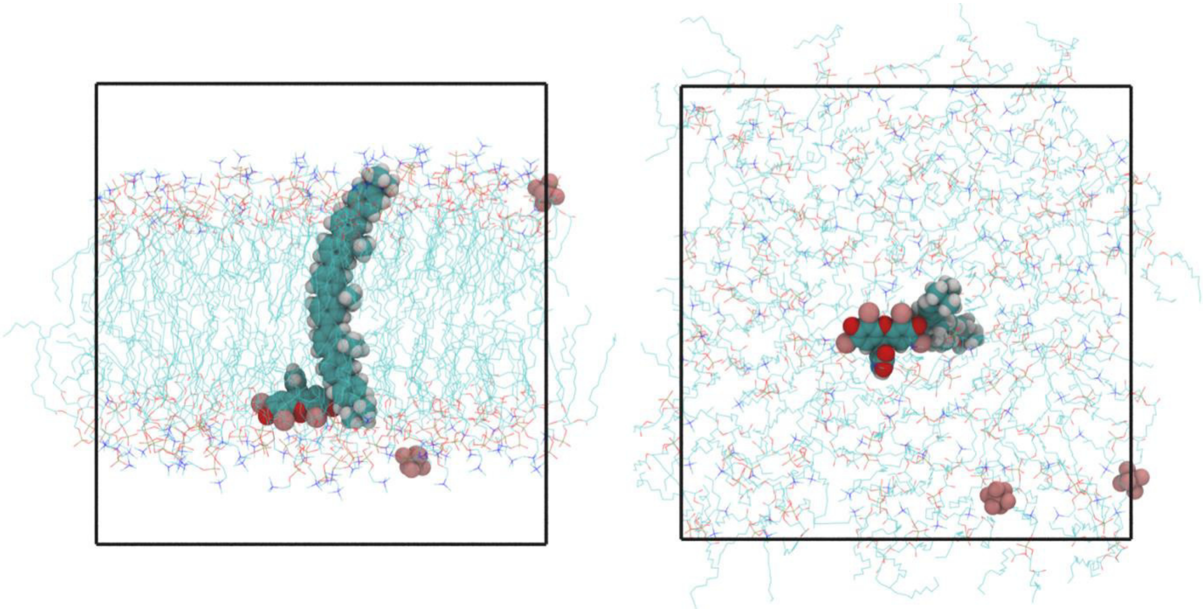

Figure S6. Orthographic projection of the EY^{2-} and $\mathbf{1}^{2+}$ complex (t = 30 ns): left for a side view, right for a top view. For visual clarity, water molecules and Na^+ ions are omitted and DMPC molecules are visualized by lines.

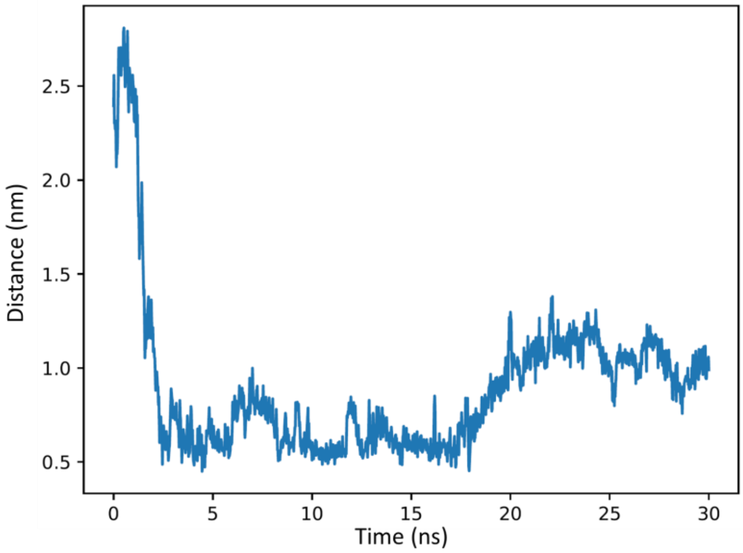

Figure S7. Temporal evolution of the distance between the positively charged N of $\mathbf{1}^{2+}$ and the center of mass of EY^{2-}.

We observed that the EY^{2-} relocated to one end of the $\mathbf{1}^{2+}$ within 2.5 ns. This association remains for the rest of the simulation time with an averaged distance of 0.87 ± 0.24 nm between the positively charged N of $\mathbf{1}^{2+}$ and the center of mass of EY^{2-}. Figure S6 shows the last snapshot at t = 30 ns and Figure S7 the evolution of the distance over time. This simulation demonstrates the possibility of the association between the EY^{2-} and $\mathbf{1}^{2+}$.

8 Optical spectra

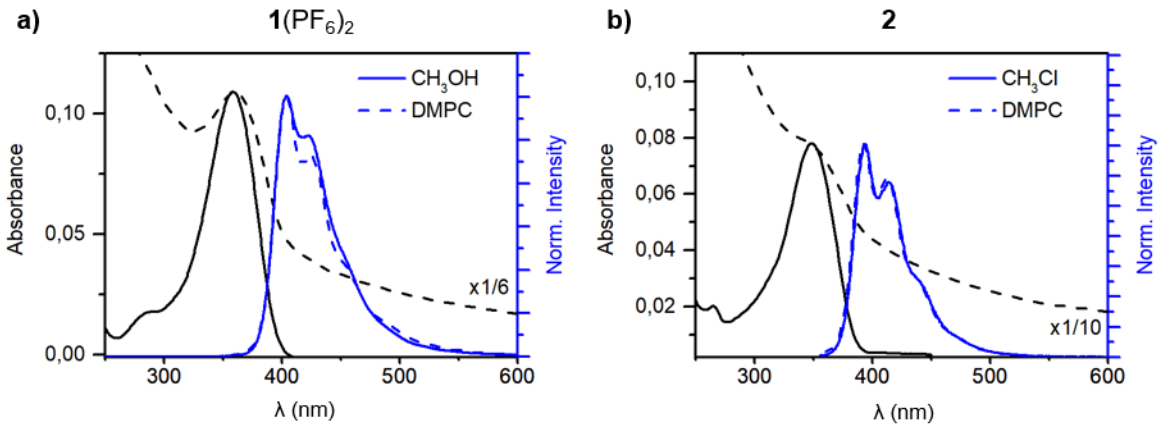

Figure S8. Absorbance and luminescence spectra of **1**(PF_6)$_2$ and **2** in organic solvent and in DMPC liposomes with 4 % NaDSPE-PEG2K and 1 % dopant (**1**(PF_6)$_2$ or **2**) in phosphate buffer, pH 7.8. Absorbance spectra of liposomes samples show the classical Tyndall scattering profile additional to the absorption band. In the graphs these liposome spectra were scaled to the absorption spectrum in organic solvent with the indicated scaling factors.

9 TD-DFT

1^{2+} and **2** were modeled in the gas phase using TD-DFT. In agreement with the experimental spectroscopic UV-vis absorption data and in agreement with the shape and position of HOMO and LUMO **1^{2+}** was modeled with CAMB3LYP (typically used for charge transfer transitions) and for **2** PB0 was applied (suitable for classical π- π* transitions).

Table S3. TD-DFT results for **1^{2+}** and **2**.

	**Model**	**λ_{Abs} (nm)**	**Transition Dipole Moment (x;y;z)**
**1^{2+}**	CAMB3LYP	352	-2.1234; 0.3999; -5.9393
**2**	PB0	353	2.0306; -4.6959; -1.2230

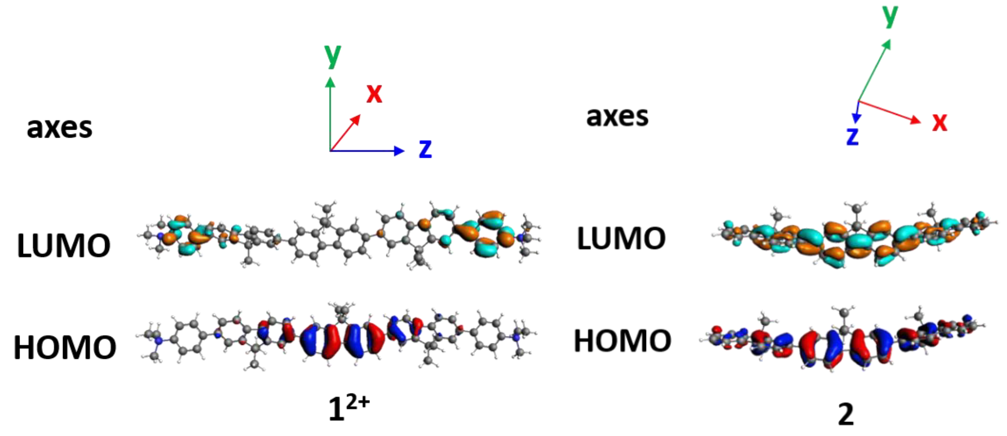

Figure S9. Graphical representation of the HOMO and LUMO of the DFT-minimized structures of **1^{2+}** and **2**.

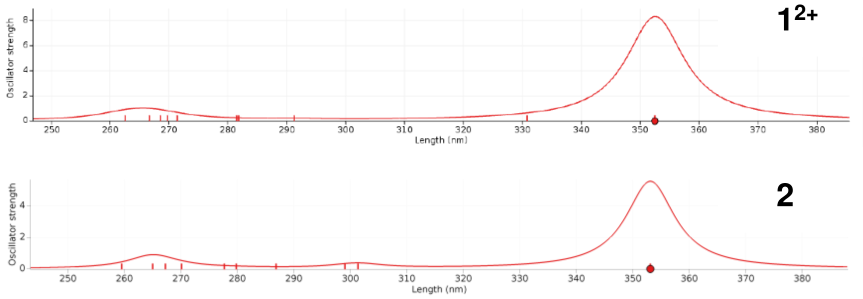

Figure S10. Calculated UV-vis absorption spectra of **1^{2+}** and **2** in the gas phase.

Table S4. Calculated atom coordinates of **1**$^{2+}$ in the ground state.

Atom	X	Y	Z (Angstrom)
1.C	-1.188678	0.930852	-4.731147
2.C	-0.120105	1.083439	-5.625094
3.C	-0.316332	1.082545	-6.994589
4.C	-1.606881	0.930102	-7.487490
5.C	-2.687035	0.785536	-6.609739
6.C	-2.482725	0.783223	-5.245239
7.C	-2.113163	0.886061	-8.851332
8.C	-3.505766	0.725901	-8.804366
9.C	-3.993457	0.633435	-7.369214
10.C	-1.456193	0.967165	-10.073474
11.C	-2.195964	0.885883	-11.240336
12.C	-3.587174	0.737934	-11.207744
13.C	-4.238860	0.661267	-9.969370
14.C	-4.643843	-0.726870	-7.092683
15.C	-4.969163	1.766652	-7.034096
16.C	-4.355019	0.671461	-12.459608
17.C	1.124126	-0.103680	3.238951
18.C	-0.081197	0.608785	3.246374
19.C	-0.711706	0.975703	2.069618
20.C	-0.130687	0.624647	0.857849
21.C	1.078142	-0.081575	0.832397
22.C	1.701569	-0.442415	2.008967
23.C	-0.567325	0.842404	-0.515187
24.C	0.376642	0.270779	-1.376634
25.C	1.521983	-0.348956	-0.594983
26.C	-1.697025	1.466966	-1.028127
27.C	-1.880943	1.497721	-2.399984
28.C	-0.955742	0.915206	-3.274563
29.C	0.186897	0.305117	-2.742699
30.C	1.645232	-1.850231	-0.873803
31.C	2.846652	0.359421	-0.903860

32.C	4.071126	-1.338291	10.965542
33.C	4.425331	-2.459427	10.206293
34.C	4.094720	-2.552332	8.865624
35.C	3.406589	-1.503777	8.266511
36.C	3.058494	-0.367732	9.011717
37.C	3.380558	-0.285782	10.348994
38.C	2.921334	-1.329626	6.905101
39.C	2.283169	-0.087176	6.818169
40.C	2.335141	0.648244	8.145770
41.C	2.989429	-2.163375	5.795394
42.C	2.408259	-1.747404	4.610936
43.C	1.759576	-0.509129	4.506933
44.C	1.709763	0.322831	5.631863
45.C	0.928763	0.940000	8.678905
46.C	3.140128	1.948536	8.029915
47.C	-3.848230	0.019923	-13.590309
48.C	-4.563342	-0.039885	-14.770180
49.C	-5.816663	0.554432	-14.847623
50.C	-6.347931	1.204201	-13.745865
51.C	-5.616941	1.257369	-12.569198
52.N	-6.564397	0.472251	-16.143594
53.C	-7.888610	1.159134	-16.086072
54.C	-6.813370	-0.965115	-16.494178
55.C	4.407787	-1.276791	12.395679
56.C	3.555880	-0.666036	13.317693
57.C	3.858040	-0.612433	14.669518
58.C	5.035235	-1.181221	15.126176
59.C	5.905853	-1.794052	14.233548
60.C	5.592724	-1.835743	12.889336
61.N	5.405986	-1.155195	16.578251
62.C	5.558820	-2.558332	17.086791
63.C	4.375951	-0.480181	17.421672
64.C	-5.763125	1.123340	-17.232485
65.C	6.697437	-0.412158	16.756431

66.H	0.879124	1.231175	-5.231851
67.H	0.527903	1.209938	-7.663561
68.H	-3.312889	0.639209	-4.561617
69.H	-0.381240	1.099557	-10.122028
70.H	-1.688812	0.976048	-12.194836
71.H	-5.314026	0.515000	-9.933202
72.H	-3.960202	-1.543749	-7.328640
73.H	-4.922847	-0.807775	-6.040291
74.H	-5.549814	-0.849577	-7.690592
75.H	-5.254902	1.722991	-5.981279
76.H	-5.880178	1.680353	-7.630976
77.H	-4.519804	2.742085	-7.226911
78.H	-0.549115	0.847407	4.194849
79.H	-1.652892	1.513551	2.103330
80.H	2.649072	-0.971312	1.991265
81.H	-2.426781	1.929287	-0.372423
82.H	-2.748737	2.004011	-2.807743
83.H	0.899091	-0.169394	-3.410015
84.H	1.900390	-2.026084	-1.921081
85.H	2.433237	-2.290906	-0.259177
86.H	0.709351	-2.367343	-0.656419
87.H	2.770960	1.431021	-0.712987
88.H	3.122665	0.214461	-1.950640
89.H	3.650465	-0.044393	-0.284506
90.H	4.932867	-3.291082	10.682759
91.H	4.362425	-3.439293	8.302652
92.H	3.128527	0.603191	10.918897
93.H	3.472167	-3.133057	5.851085
94.H	2.424060	-2.407647	3.751523
95.H	1.234160	1.294987	5.553925
96.H	0.401666	1.620623	8.007517
97.H	0.343185	0.023465	8.766248
98.H	0.979056	1.414993	9.661472
99.H	3.223990	2.437558	9.003157

100.H	2.645466	2.641981	7.347094
101.H	4.145209	1.755396	7.652235
102.H	-2.884081	-0.471373	-13.537828
103.H	-4.127985	-0.564949	-15.612401
104.H	-7.313844	1.688443	-13.770755
105.H	-6.031978	1.795031	-11.725249
106.H	-8.507274	0.689018	-15.325675
107.H	-8.359858	1.051493	-17.060836
108.H	-7.740082	2.213142	-15.864348
109.H	-7.396921	-1.416094	-15.694958
110.H	-5.863230	-1.481244	-16.592788
111.H	-7.358967	-1.003254	-17.435605
112.H	2.616027	-0.245439	12.981288
113.H	3.151169	-0.135645	15.333672
114.H	6.840373	-2.235190	14.560095
115.H	6.295467	-2.293340	12.203619
116.H	5.820239	-2.518128	18.143054
117.H	6.342368	-3.060354	16.527514
118.H	4.613856	-3.077851	16.945297
119.H	3.432452	-1.013721	17.336635
120.H	4.721600	-0.508088	18.452944
121.H	4.264254	0.552390	17.100256
122.H	-5.590496	2.160834	-16.955505
123.H	-6.326953	1.062898	-18.161940
124.H	-4.813761	0.607214	-17.338237
125.H	7.479859	-0.911296	16.192828
126.H	6.562800	0.600539	16.383580
127.H	6.948192	-0.400751	17.815941

Table S5. Calculated atom coordinates of **2** in the ground state.

Atom	X	Y	Z (Angstrom)
1.C	-4.585450	-1.636752	1.213974
2.C	-5.375433	-1.999808	2.311517
3.C	-6.744634	-2.176637	2.192763
4.C	-7.344832	-1.988355	0.954090
5.C	-6.570241	-1.621658	-0.151979
6.C	-5.206860	-1.447880	-0.026674
7.C	-8.736577	-2.106177	0.526964
8.C	-8.805146	-1.808775	-0.839865
9.C	-7.430791	-1.479804	-1.395228
10.C	-9.882177	-2.446776	1.236060
11.C	-11.097324	-2.485363	0.565959
12.C	-11.166826	-2.187255	-0.791449
13.H	-12.123658	-2.221254	-1.300568
14.C	-10.019290	-1.846687	-1.501451
15.C	-7.377507	-0.051343	-1.948186
16.C	-7.015901	-2.482207	-2.478132
17.C	3.564227	0.279992	0.962972
18.C	3.419289	-0.785755	1.859156
19.C	2.177881	-1.335511	2.136198
20.C	1.052609	-0.811982	1.511599
21.C	1.180053	0.259794	0.619909
22.C	2.420057	0.799400	0.345076
23.C	-0.357058	-1.178478	1.595034
24.C	-1.086900	-0.326368	0.757586
25.C	-0.175098	0.680241	0.078249
26.C	-1.009054	-2.169003	2.318767
27.C	-2.382425	-2.302528	2.192025
28.C	-3.126188	-1.462572	1.354305
29.C	-2.454815	-0.463641	0.638300
30.C	-0.517187	2.113039	0.503052
31.C	-0.235769	0.551096	-1.447019

32.C	10.645676	3.569122	-2.005542
33.H	11.440524	3.991533	-2.610218
34.C	10.840650	3.389121	-0.639438
35.C	9.835930	2.850499	0.152693
36.C	8.631925	2.494681	-0.443024
37.C	8.434613	2.675331	-1.817813
38.C	9.439934	3.211877	-2.601939
39.C	7.415697	1.917905	0.124291
40.C	6.479826	1.754467	-0.903106
41.C	7.049583	2.211756	-2.234679
42.C	7.097965	1.542267	1.423332
43.C	5.846068	1.007879	1.682803
44.C	4.896556	0.843363	0.667501
45.C	5.232614	1.226290	-0.636651
46.C	6.229104	3.361725	-2.828021
47.C	7.131171	1.049938	-3.231271
48.H	-4.906445	-2.120825	3.281679
49.H	-7.332575	-2.450807	3.062205
50.H	-4.602065	-1.193849	-0.891412
51.H	-9.833083	-2.680221	2.294350
52.H	-12.000946	-2.750532	1.103743
53.H	-10.085963	-1.618013	-2.560515
54.H	-8.055656	0.056955	-2.797699
55.H	-7.664499	0.673645	-1.184778
56.H	-6.368005	0.189196	-2.289690
57.H	-7.682202	-2.411050	-3.340873
58.H	-5.998135	-2.279522	-2.819273
59.H	-7.053725	-3.504372	-2.098274
60.H	4.304483	-1.207419	2.322596
61.H	2.096309	-2.170115	2.824441
62.H	2.517968	1.645079	-0.328600
63.H	-0.457110	-2.842615	2.965602
64.H	-2.891115	-3.095305	2.729185
65.H	-3.023793	0.213168	0.008727

66.H	0.187627	2.821578	0.062199
67.H	-1.521951	2.382084	0.169459
68.H	-0.476054	2.217775	1.588264
69.H	-1.235902	0.794488	-1.812902
70.H	0.470812	1.238062	-1.918420
71.H	0.008279	-0.464494	-1.763037
72.H	11.786312	3.672834	-0.190781
73.H	9.993036	2.712518	1.217122
74.H	9.299519	3.356185	-3.668566
75.H	7.810457	1.670421	2.231235
76.H	5.582569	0.736036	2.699134
77.H	4.513492	1.075732	-1.435731
78.H	5.216245	3.027230	-3.064232
79.H	6.161745	4.195449	-2.127205
80.H	6.688032	3.724114	-3.750640
81.H	6.132014	0.679098	-3.470603
82.H	7.602970	1.375535	-4.161084
83.H	7.714775	0.224452	-2.820774

10 Liposome Stability Tests

Liposomes of DPPC with 1 % NaDSPE-PEG2K and optionally 10 % **1**$(PF_6)_2$ were prepared as described above. Lipid film rehydration was carried out with a phosphate buffer (100 mM, pH 7.7), containing 70 mM calcein. After extrusion, the liposomes were separated from the not encapsulated calcein using a GE Healthcare Illustra™ NAP™-25 size exclusion chromatography cartridges equilibrated with 100 mM phosphate buffer. Liposomes eluted from the column contained a self-quenching concentration of calcein. These liposome stock solutions were stored at RT and samples were analyzed with DLS and calcein luminescence over the course of one week.

Dynamic light scattering

Dynamic light scattering monitoring these samples over the course of one week of storage at room temperature was performed at 25 °C. The respective data below show the hydrodynamic diameter (Z_{Avg}), and polydispersity index (PDI) of all samples, showing that the liposomes remain stable in size (around 165 d.nm) with a constant PDI of around 0.1, which is considered as monodisperse.

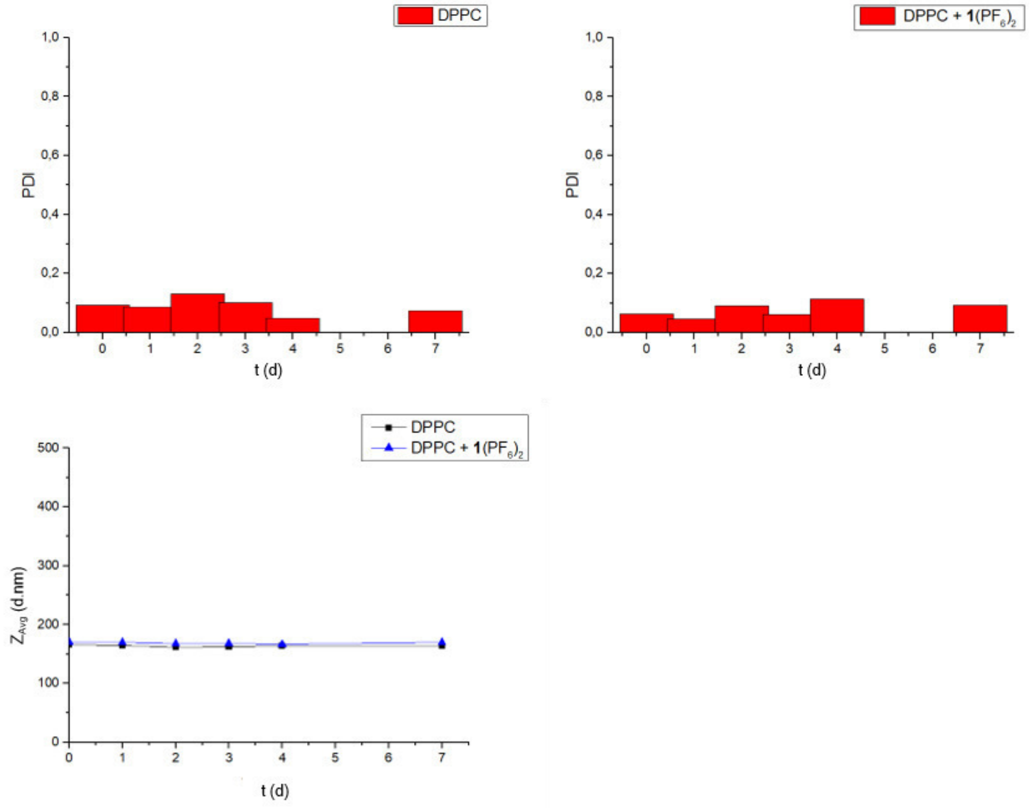

Figure S11. Dynamic light scattering data in 100 mM pH 7.7 phosphate buffer of DPPC liposomes with 1 % NaDSPE-PEG2K and DPPC liposomes with 1 % NaDSPE-PEG2K and with or without 10 % **1**$(PF_6)_2$.

Calcein-luminescence leakeage test

In a fluorescence quartz cuvette with 2970 µL of phosphate buffer, 30 µL of the liposome mixture was added (100x dilution). A luminescence measurement was carried out with excitation at λ_{ex} = 495 nm and by recording the corresponding emission spectrum. Next, Triton X-100 solution (1.5 mM, 500 µL) in the previously prepared phosphate buffer was added to the cuvette and mixed. After 10 min, the solution was measured again at the same conditions. This process induces the destruction of the liposomal membrane and releases the calcein into the bulk solution.[24] Because this yields calcein at lower, not-self-quenching concentration, the luminescence intensity is massively increased. The maximum luminescence intensity at 518 nm was used to determine the increase of calcein leakeage during the storage as compared to purposeful membrane destruction with Triton X-100.

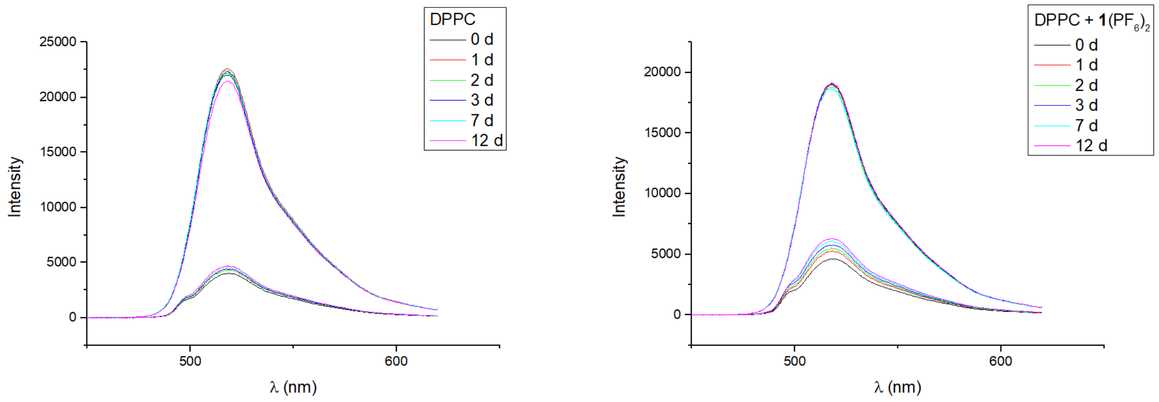

Figure S12. Luminescence Spectra of calcein in DPPC liposomes with 1 % NaDSPE-PEG2K and with or without 10 % **1**$(PF_6)_2$ at self-quenching concentration (low intensity) and spectra upon destruction of the lipid membrane (high intensity) at all time points. Excitation occurred at 495 nm.

Table S6. Increase of calcein luminescence prior to treatment with Triton X with respect to maximum luminescence at λ_{max} = 518 nm after liposomal membrane destruction.

	**Calcein luminescence increase (%)**	
**t (d)**	**DPPC**	**DPPC + 1(PF_6)$_2$**
0	0.0	0.0
1	1.3	4.6
2	1.9	4.5
3	2.7	5.9
7	3.4	5.0
12	4.8	9.0

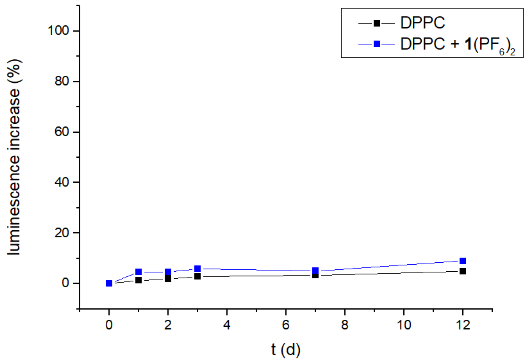

Figure S13. Increase of calcein luminescence prior to treatment with Triton X with respect to the maximum luminescence at λ_{max} = 518 nm after liposomal membrane destruction.

9 Stern-Volmer quenching experiment

Liposomes were prepared as described above.

In cuvette:

V = 3 mL

c(**1**$^{2+}$) = 5 µM

c(DPPC) = 400 µM

c(NaDSPE-PEG2K) = 1 µM

c(EY^{2-}) = [0;4] µM

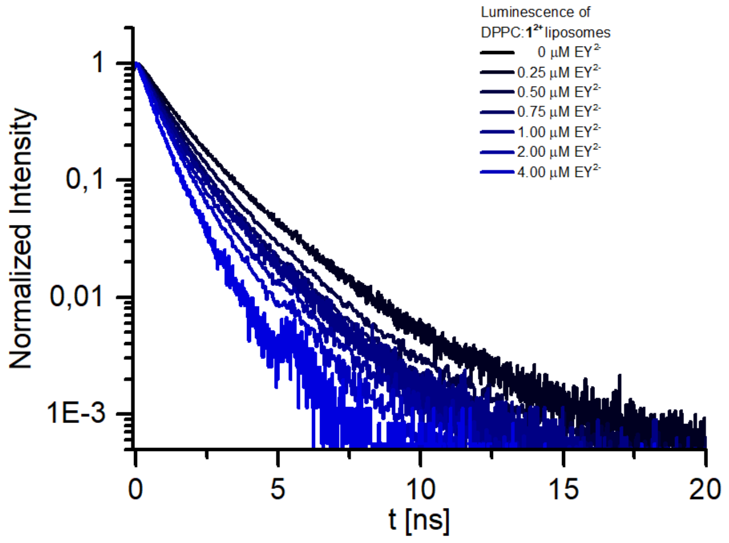

Figure S14. Kinetic traces of luminescence decay upon excitation at 375 nm at pH 7.8 of DPPC liposomes with 0.3 % NaDSPE-PEG2K, 1.3 % **1**(PF_6)$_2$ and various concentrations of EY^{2-} that were added to lipid film during its preparation. Concentration values are reported for the final samples during the spectroscopic experiment.

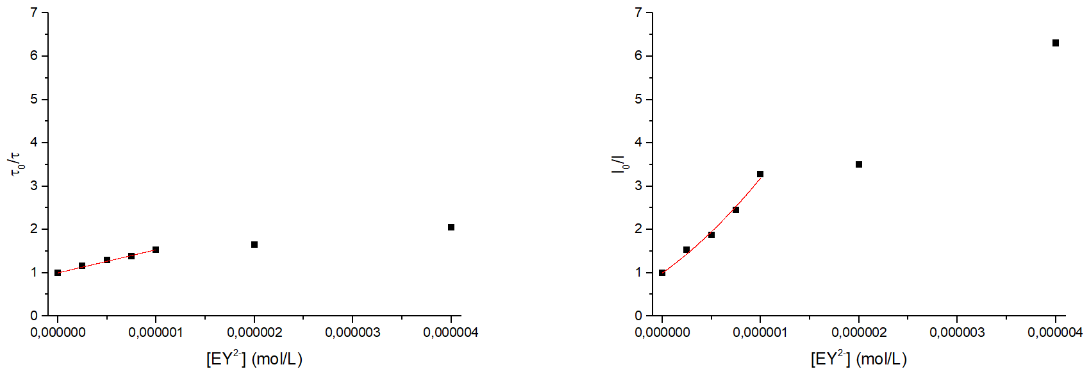

Figure S15. Luminescence quenching data.

10 Confocal microscopy

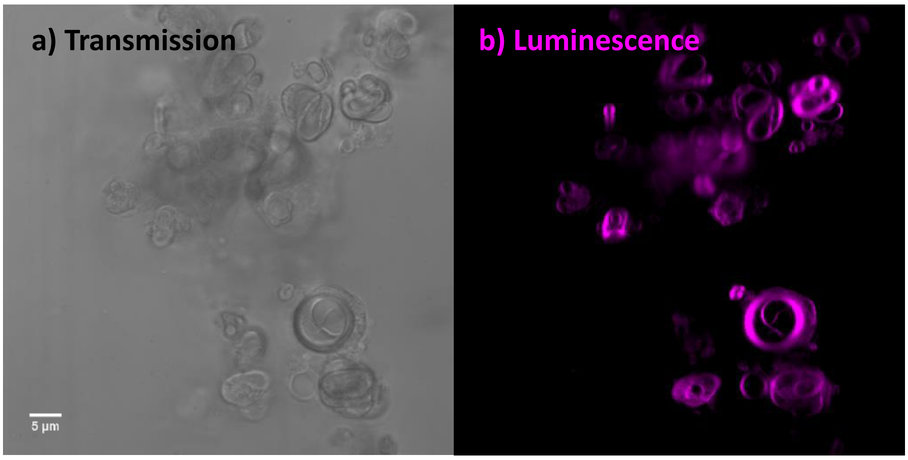

Figure S16. Confocal microscopy images of giant DMPC vesicles doped with 1 mol-% $\mathbf{1}^{2+}$ at pH 7.8, laser excitation at λ_{ex} = 405 nm, a) Transmission, b) Luminescence images λ_{det} = [420 – 514 nm].

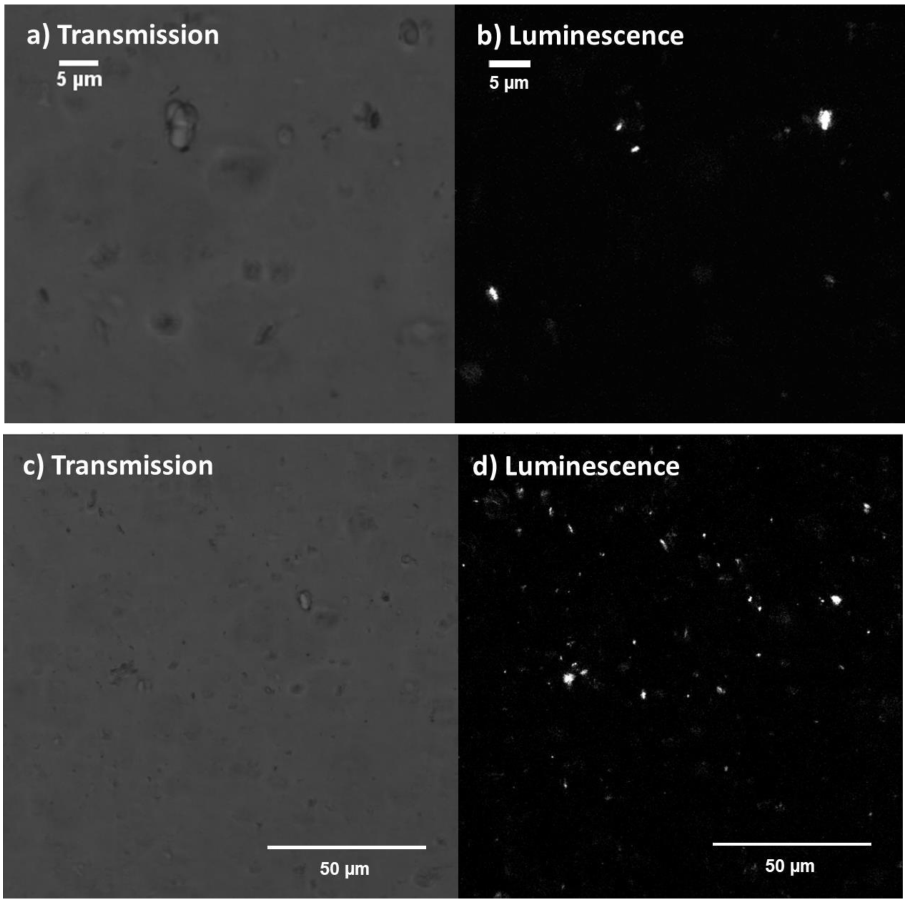

Figure S17. Confocal microscopy image of DMPC vesicles doped with 7 mol-% **2** at pH 7.8, laser excitation at λ_{ex} = 405 nm at two different zoom levels with indicated scale. a,c) Transmission, b,d) Luminescence images λ_{det} = [420 – 514 nm]. Experimental note: During vesicle preparation, additional sonication for five minutes was applied at 30 °C in presence of buffer to form smaller structures. It seems that only few larger vesicle-like structures have formed, but without membrane staining by **2**. Under identical experimental conditions, the identical sample preparation was performed for $\mathbf{1}^{2+}$ yielding membrane-stained vesicles comparable to the ones shown in Figure S15, but smaller.

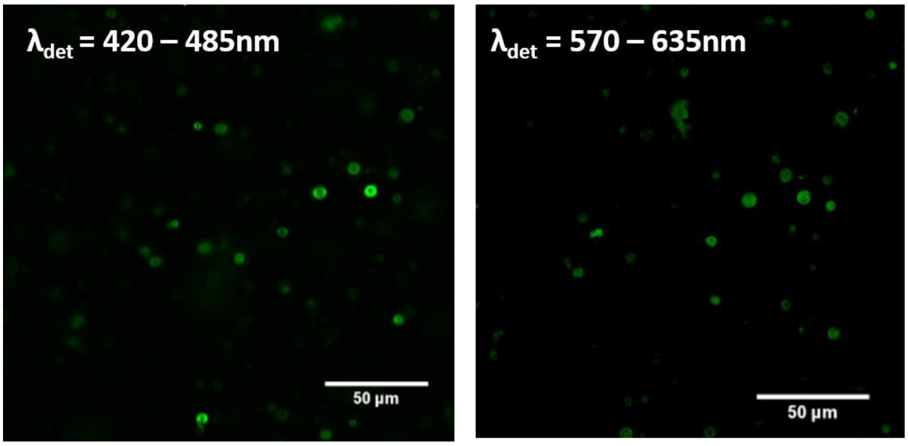

Figure S18. c) Confocal images (excitation at 405 nm) of DPPC:$\mathbf{1}^{2+}$ in presence of 10 µM EY^{2-} added to the solution after vesicle formation at pH 7.8.

11 Eosin Y species and EDTA

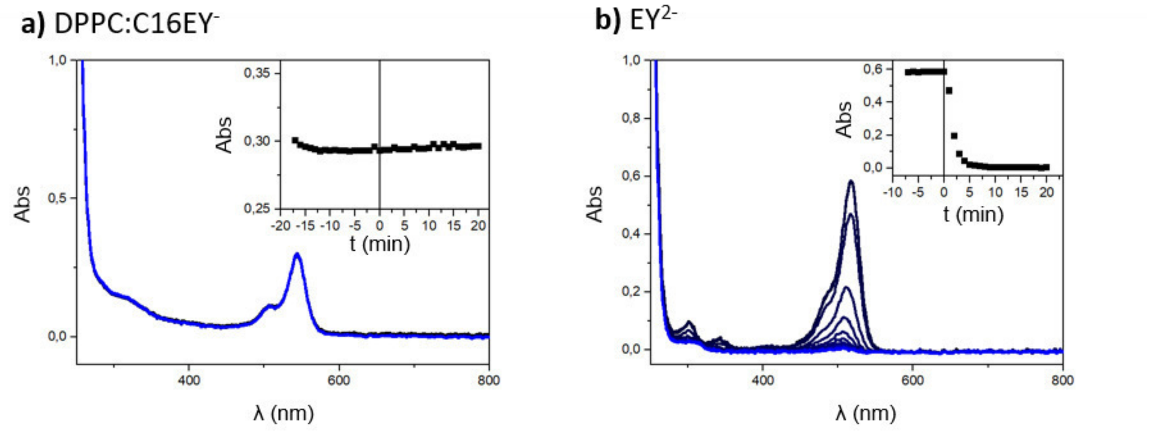

Figure S19. Evolution of UV-vis absorption spectra of eosin Y species in presence of $EDTA^{4-}$ (42 mM) at pH 7.8 upon irradiation with 375 nm LED (0.5 mW) and kinetic traces at the absorption maximum. a) DPPC:$C16EY^{-}$ liposomes at 0.5 mM DPPC concentration with 1 % $C16EY^{-}$ and 1 % NaDSPE-PEG2K added to the lipid film during liposome preparation. b) 6.7 μM EY^{2-}.

12 References

[1] E. Krieger, J. E. Nielsen, C. A. E. M. Spronk, G. Vriend, *J. Mol. Graph. Model.* **2006**, *25*, 481–486.

[2] E. Krieger, T. Darden, S. B. Nabuurs, A. Finkelstein, G. Vriend, *Proteins Struct. Funct. Bioinforma.* **2004**, *57*, 678–683.

[3] E. Krieger, G. Vriend, *J. Comput. Chem.* **2015**, *36*, 996–1007.

[4] J. Srinivasan, T. E. Cheatham, P. Cieplak, P. A. Kollman, D. A. Case, *J. Am. Chem. Soc.* **1998**, *120*, 9401–9409.

[5] R. Kumari, R. Kumar, A. Lynn, *J. Chem. Inf. Model.* **2014**, *54*, 1951–1962.

[6] R. Ma, S. W. Wong, L. Ge, C. Shaw, S. W. I. Siu, H. F. Kwok, *Mol. Ther. - Oncolytics* **2020**, *16*, 7–19.

[7] J. Zhang, H. Zhang, T. Wu, Q. Wang, D. Van Der Spoel, *J. Chem. Theory Comput.* **2017**, *13*, 1034–1043.

[8] M. J. Abraham, T. Murtola, R. Schulz, S. Páll, J. C. Smith, B. Hess, E. Lindah, *SoftwareX* **2015**, *1–2*, 19–25.

[9] J. A. Maier, C. Martinez, K. Kasavajhala, L. Wickstrom, K. E. Hauser, C. Simmerling, *J. Chem. Theory Comput.* **2015**, *11*, 3696–3713.

[10] A. W. Sousa da Silva, W. F. Vranken, *BMC Res. Notes* **2012**, *5*, 367.

[11] W. D. Cornell, P. Cieplak, C. I. Bayly, P. A. Kollman, *J. Am. Chem. Soc.* **1993**, *115*, 9620–9631.

[12] M. J. Frisch, G. W. Trucks, H. B. Schlegel, G. E. Scuseria, M. a. Robb, J. R. Cheeseman, G. Scalmani, V. Barone, G. a. Petersson, H. Nakatsuji, X. Li, M. Caricato, a. V. Marenich, J. Bloino, B. G. Janesko, R. Gomperts, B. Mennucci, H. P. Hratchian, J. V. Ortiz, a. F. Izmaylov, J. L. Sonnenberg, Williams, F. Ding, F. Lipparini, F. Egidi, J. Goings, B. Peng, A. Petrone, T. Henderson, D. Ranasinghe, V. G. Zakrzewski, J. Gao, N. Rega, G. Zheng, W. Liang, M. Hada, M. Ehara, K. Toyota, R. Fukuda, J. Hasegawa, M. Ishida, T. Nakajima, Y. Honda, O. Kitao, H. Nakai, T. Vreven, K. Throssell, J. a. Montgomery Jr., J. E. Peralta, F. Ogliaro, M. J. Bearpark, J. J. Heyd, E. N. Brothers, K. N. Kudin, V. N. Staroverov, T. a. Keith, R. Kobayashi, J. Normand, K. Raghavachari, a. P. Rendell, J. C. Burant, S. S. Iyengar, J. Tomasi, M. Cossi, J. M. Millam, M. Klene, C. Adamo, R. Cammi, J. W. Ochterski, R. L. Martin, K. Morokuma, O. Farkas, J. B. Foresman, D. J. Fox, *Gaussian 16, Revision C.01*, Gaussian, Inc., Wallingford CT, **2016**.

[13] J. P. M. Jämbeck, A. P. Lyubartsev, *J. Phys. Chem. B* **2012**, *116*, 3164–3179.

[14] W. L. Jorgensen, J. Chandrasekhar, J. D. Madura, R. W. Impey, M. L. Klein, *J. Chem. Phys.* **1983**, *79*, 926–935.

[15] G. Bussi, D. Donadio, M. Parrinello, *J. Chem. Phys.* **2007**, *126*, 014101.

[16] H. J. C. Berendsen, J. P. M. Postma, W. F. Van Gunsteren, A. Dinola, J. R. Haak, *J. Chem. Phys.* **1984**, *81*, 3684–3690.

[17] M. Parrinello, A. Rahman, *J. Appl. Phys.* **1981**, *52*, 7182–7190.

[18] S. Nosé, M. L. Klein, *Mol. Phys.* **1983**, *50*, 1055–1076.

[19] U. Essmann, L. Perera, M. L. Berkowitz, T. Darden, H. Lee, L. G. Pedersen, *J. Chem. Phys.* **1995**, *103*, 8577–8593.

[20] M. Sudhakar, P. I. Djurovich, T. E. Hogen-Esch, M. E. Thompson, *J. Am. Chem. Soc.* **2003**, *125*, 7796–7797.

[21] V. Novakova, M. Miletin, K. Kopecky, P. Zimcik, *Chem. - A Eur. J.* **2011**, *17*, 14273–14282.

[22] J. Odrobina, J. Scholz, A. Pannwitz, L. Francàs, S. Dechert, A. Llobet, C. Jooss, F. Meyer, *ACS Catal.* **2017**, *7*, 2116–2125.

[23] S. S. S. Tan, P. C. Hauser, K. Wang, K. Fluri, K. Seiler, B. Rusterholz, G. Suter, M. Krüttli, U. E. Spichiger, W. Simon, *Anal. Chim. Acta* **1991**, *255*, 35–44.

[24] B. Limburg, E. Bouwman, S. Bonnet, *Chem. Commun.* **2015**, *51*, 17128–17131.
